# Bioassay-Guided Isolation and Structure Elucidation of Fungicidal and Herbicidal Compounds from *Ambrosia salsola* (Asteraceae)

**DOI:** 10.3390/molecules24050835

**Published:** 2019-02-26

**Authors:** Wilmer H. Perera, Kumudini M. Meepagala, Frank R. Fronczek, Daniel D. Cook, David E. Wedge, Stephen O. Duke

**Affiliations:** 1 ORISE Fellow-Agricultural Research Service, U.S. Department of Agriculture, Natural Products Utilization Research Unit, Texas A&M University, P.O. Box 1848, Oxford, MS 38677, USA; 2 Agricultural Research Service, U.S. Department of Agriculture, Natural Products Utilization Research Unit, Texas A&M University, P.O. Box 1848, Oxford, MS 38677, USA; david.wedge@ars.usda.gov (D.E.W.); Stephen.Duke@ars.usda.gov (S.O.D.); 3 Department of Chemistry, Louisiana State University, Baton Rouge, LA 70803, USA; ffroncz@lsu.edu; 4 Agricultural Research Service, U.S. Department of Agriculture, Poisonous Plant Research Lab. 1150 E. 1400 N., Logan, UT 84341, USA; Daniel.Cook@ars.usda.gov

**Keywords:** *Ambrosia salsola*, *Ambrosia dumosa*, β-Hydroxydihydrochalcones, fungitoxic and phytotoxic compounds, confertin, X-ray diffraction

## Abstract

The discovery of potent natural and ecofriendly pesticides is one of the focuses of the agrochemical industry, and plant species are a source of many potentially active compounds. We describe the bioassay-guided isolation of antifungal and phytotoxic compounds from the ethyl acetate extract of *Ambrosia salsola* twigs and leaves. With this methodology, we isolated and identified twelve compounds (four chalcones, six flavonols and two pseudoguaianolide sesquiterpene lactones). Three new chalcones were elucidated as (*S*)-β-Hydroxy-2′,3,4,6′-tetrahydroxy-5-methoxydihydrochalcone (salsolol A), (*S*)-β-Hydroxy-2′,4,4′,6′-tetrahydroxy-3-methoxydihydrochalcone (salsolol B), and (*R*)-α, (*R*)-β-Dihydroxy-2′,3,4,4′,6′-pentahydroxydihydrochalcone (salsolol C) together with nine known compounds: balanochalcone, six quercetin derivatives, confertin, and neoambrosin. Chemical structures were determined based on comprehensive direct analysis in real time-high resolution mass spectrometry (HR-DART-MS), as well as 1D and 2D NMR experiments: Cosy Double Quantum Filter (DQFCOSY), Heteronuclear Multiple Quantum Coherence (HMQC) and Heteronuclear Multiple Bond Coherence (HMBC), and the absolute configurations of the chalcones were confirmed by CD spectra analysis. Crystal structure of confertin was determined by X-ray diffraction. The phytotoxicity of purified compounds was evaluated, and neoambrosim was active against *Agrostis stolonifera* at 1 mM, while confertin was active against both, *Lactuca sativa* and *A. stolonifera* at 1 mM and 100 µM, respectively. Confertin and salsolol A and B had IC_50_ values of 261, 275, and 251 µM, respectively, against *Lemna pausicotata* (duckweed). The antifungal activity was also tested against *Colletotrichum fragariae* Brooks using a thin layer chromatography bioautography assay. Both confertin and neoambrosin were antifungal at 100 µM, with a higher confertin activity than that of neoambrosin at this concentration.

## 1. Introduction

The annual global human population has been expanding remarkably for more than a century. To sustain this growth, crop productivity needs to be increased. However, considerable crop losses occur each year due to weeds, insects, and plant pathogens, causing billions of dollars in economic damage. Enhanced crop pest resistance and the use of synthetic pesticides have been the main approaches implemented for overcoming crop pests. Despite increasingly stringent regulation of pesticides, there is still concern regarding undesirable health and environmental effects [1,2]. Natural products are perceived to be sources of safer pesticides. Resistance to synthetic pesticides is rapidly evolving, and pesticides with new modes of action are needed for pesticide resistance management. Natural products are a source of compounds with new modes of action [3]. In recent years, only a small percentage of approved new pesticide active ingredients in the United States of America have been based on natural products [3,4].

Plant species have historically been a valuable source of biologically active compounds, or templates for the synthesis of more potent bioactive candidates. Bioassay-guided isolation is the most common strategy for screening a large pool of active plant extracts and fractions to find active principles [5,6]. The genus *Ambrosia* L. is comprised of more than 40 species and belongs to one of the largest botanical families, Asteraceae. Species, or compounds isolated from this genus, have been reported for their allelopathic, anti-inflammatory, and antiprotozoal activities [7,8,9,10,11]. *Ambrosia salsola* (Torr. & A. Gray) Strother & B. G. Baldw (synonym *Hymenoclea salsola* Torr. & A. Gray), a species that thrives in desert environments in some areas of the USA, was chosen for the discovery of phytotoxic and fungitoxic compounds. Few chemical reports have been published regarding the purification of secondary metabolites from *A. salsola*. Some pseudoguaianolide sesquiterpene lactones have been purified, and some flavonoids were detected by thin layer chromatography from leaf resins from this species [12,13,14]. Herein, we report the isolation and structure elucidation of four hydroxydihydrochalcones, including three new chemical structures together with six flavonols and two pseudoguaianolide sesquiterpene lactones from the ethyl acetate extract of *A. salsola*. Pure compounds were evaluated for antifungal and phytotoxic activity.

## 2. Results and Discussion

### 2.1. Structure Elucidation

Leaves and twigs of *A. salsola* collected in Texas were extracted sequentially with hexane, ethyl acetate, and methanol. Phytotoxicity and antifungal bioassays allowed us to identify active fractions. The ethyl acetate extract was phytotoxic and antifungal in the bioassays. Therefore, ethyl acetate extract was fractionated by silica gel flash chromatography to produce 14 main fractions. Further fractionation and purification from the most active fractions led to the isolation and characterization of 12 compounds.

Compound **1** was purified as a yellowish powder, [α]^25^_D_ −20.0 (c 0.1, MeOH). Its UV spectrum absorption maxima at 225 and 288 nm revealed a β-hydroxydihydrochalcone skeleton [15]. The molecular formula was established as C_16_H_15_O_6_ by HR-DART-MS (comprehensive direct analysis in real time-high resolution mass spectrometry) positive mode ion, which showed a quasi-molecular ion [M + H−H_2_O]^+^ at 303.0869 (Calcd for C_16_H_15_O_6_, 303.0869).

2D-HMQC (Multiple Quantum Coherence) spectrum showed the presence of five aromatic signals (δ_H_ 5.87; δ_C_ 96.4 to 6.95 ppm; δ_C_ 114.7 ppm), one carbinol (δ_H_ 5.31 ppm; δ_C_ 80.4 ppm), one methylene (δ_H_ 2.71; 3.05 ppm; δ_C_ 44.2 ppm), one methoxy group (δ_H_ 3.86 ppm and δ_C_ 56.6 ppm), and one conjugate ketone at 197.7 ppm.

DQFCOSY (Cosy Double Quantum Filter) exhibited two aromatic spin systems through correlation between δ_H_ 5.87 and 5.89 ppm, and δ_H_ 6.95 and 6.91 ppm, respectively. Both protons from the A ring showed small coupling constants *J*~2.0 Hz. HMBC (Heteronuclear Multiple Bond Coherence) correlations of δ_H_ 5.89 with δ_C_ 103.5; 164.9 and 168.7 ppm together with δ_H_ 5.87 with δ_C_ 103.5; 165.6 and 168.7 ppm confirmed positions of protons in A ring δ_H_ 5.89/δ_C_ 97.3 (H-3′/C-3′) and δ_H_ 5.87/δ_C_ 96.4 (H-5′/C-5′).

**Figure 1:**
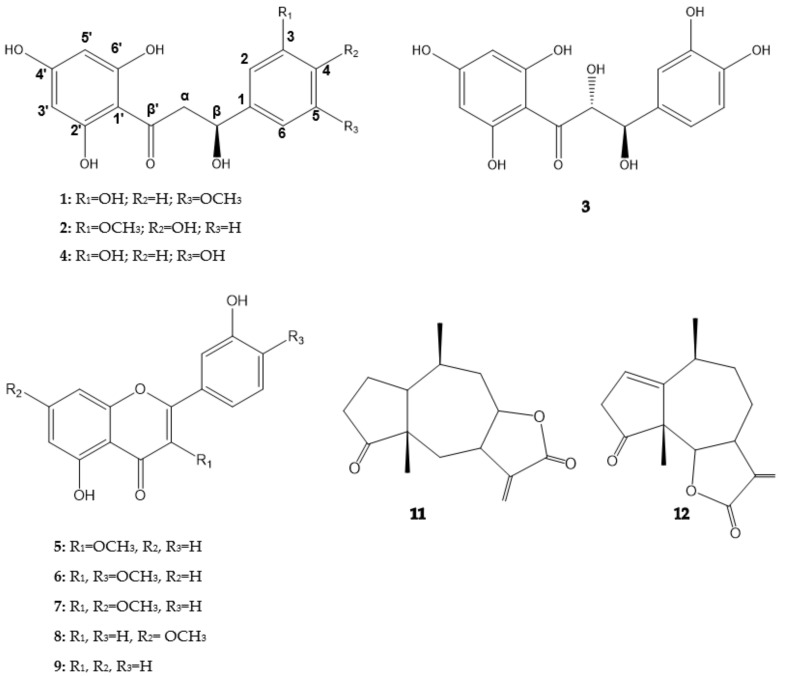
Chemical structures of purified metabolites from ethyl acetate extract from *Ambrosia salsola*: Salsolol A (**1**), salsolol B (**2**), salsolol C (**3**), balanochalcone (**4**), quercetin 3-methylether (**5**), quercetin 3,4′-methylether (**6**), quercetin 3,7-dimethylether (**7**), quercetin 7-methylether (**8**), quercetin (**9**), quercetin 3,4′,7-trimethylether (**10**), confertin (**11**), and neoambrosin (**12**).

Protons from the B ring showed a similar pattern to that recently reported for balanochalcone, one singlet at δ_H_ 6.95 (1H) and a doublet with a small coupling constant at δ_H_ 6.91 (2H) [15]. The difference observed was the presence of a methoxy group in the B ring. HMBC correlations of δ_H_ 6.95 with 80.4, 119.1, and 147.9 ppm, and δ_H_ 6.91 with 133.3 and 149.5 ppm corroborated the positions C-3 and C-5 of the hydroxyl and the methoxy groups, respectively. Carbinol, methylene, and ketone groups were found to be attached at positions C*β*, C*α*, and C*β*′, respectively. Proton and carbon NMR chemical shifts are presented in Table 1. The absolute configuration of the β stereocenter was deduced through circular dichroism (CD) spectrum. The CD spectrum showed a positive [*θ*]_328_ +8361 and a negative cotton effect [*θ*]_303_ −3829 (Supplementary Materials) similar to those reported for *β*-Hydroxydihydrochalcones containing β carbons with an S configuration [16]. Compound **1** was found to be a new chalcone with an (*S*) configuration at the β carbon, named (*S*)-β-Hydroxy-2′,3,4′,6′-tetrahydroxy-5-methoxydihydrochalcone (salsolol A). The chemical structure of compound **1** is shown in Figure 1.

Compound **2** was purified as a pale-yellow powder; [α]^25^_D_ −12.1 (c 0.1, MeOH). Its UV spectrum had absorption maxima at 225 and 288 nm and was similar to **1**, indicating the same β-hydroxydihydrochalcone skeleton. The molecular formula was established as C_16_H_15_O_6_ by HR-DART-MS positive mode, which showed a quasi-molecular ion [M + H−H_2_O]^+^ at 303.0869 (Calcd for C_16_H_15_O_6_, 303.0869). 2D-HMQC exhibited the presence of five aromatic signals (δ_H_ 5.87 to 7.04 ppm; δ_C_ 103.2 to 169.8 ppm), one carbinol (δ_H_ 5.34 ppm; δ_C_ 80.8 ppm), one methylene (δ_H_ 2.70; 3.12 ppm and δ_C_ 44.3 ppm), one methoxy group (δ_H_ 3.88 ppm and δ_C_ 56.7 ppm), and one conjugate ketone at 197.6 ppm. Protons from the A ring showed a similar profile for those found in compound **1.** Coupling constants and ^3^*J* HMBC correlations confirmed positions of protons in the A ring at δ_H_ 5.89/δ_C_ 97.6 (H-3′/C-3′) and δ_H_ 5.87/δ_C_ 96.1 (H-5′/C-5′), as seen in Table 1.

DQFCOSY showed correlation between δ_H_ 6.82, 6.92, and 7.07 ppm. These protons were assigned to the B ring because of the dual ^3^*J* HMBC correlations between δ_H_ 5.34 with 111.5 and 120.7 ppm, and 6.93/7.07 ppm with the carbinol carbon (80.8 ppm). In the same way, the carbinol group was found to be located at the C*β* position. HMBC also confirmed the presence of a methylene group at position C*α* and a ketone group at C*β*′. Additionally, protons from the B ring exhibited a typical ABX substitution pattern, and ^3^*J* HMBC showed a cross peak for the δ_H_ 3.88 ppm with δ_C_ 149.3 ppm. Thus, the position of the methoxy group was directly confirmed from the ^3^*J* HMBC correlation between δ_H_ 6.82 ppm with δ_C_ 149.3 ppm, and indirectly through ^3^*J* HMBC correlations between δ_H_ 6.93 ppm and δ_H_ 7.07 ppm with δ_C_ 148.3 ppm (C-4). The CD spectrum of **2** ([*θ*]_329_ +640, [*θ*]_298_ −1392) was similar to that obtained for **1** (Supplementary Materials). Thus, the *β* carbon of compound **2** was also assigned an (*S*) configuration and named as (*S*)-β-Hydroxy-2′,4,4′,6′-tetrahydroxy-3-methoxydihydrochalcone (salsolol B). The chemical structure is shown in Figure 1.

Compound **3** was purified as an amorphous brownish powder, [α]^25^_D_ +10.0 (c 0.1, MeOH). Its UV spectrum indicated that **3** was an analogue of previously described compounds. The molecular formula was established as C_15_H_13_O_7_ by HR-DART-MS positive mode, which showed a quasi-molecular ion [M + H−H_2_O]^+^ at 305.0661 (Calcd for C_15_H_13_O_7_, 305.0662). MS and NMR data of compound **3** matched with those reported for cilicicone b, previously isolated from *Thymus cilicicus* Linn. (Labiatae), and also found in *Toxicodendron vernicifluum* (Stokes) F.A. Barkley (formerly *Rhus verniciflua* Stokes) (Anacardiaceae) with descriptions of the absolute configuration [17,18]. β-Hydroxydihydrochalcones are a scarce subclass of flavonoids with few natural examples described, while α- and β-dihydroxyhydrochalcones are still less common. Coupling constants calculated for the α and β protons were similar to those reported for cilicicone b, however, the CD spectrum of **3** showed a positive and a negative Cotton effect, similar to previous compounds ([*θ*]_330_ +6574, [*θ*]_298_ −8028] (Supplementary Materials), but different from the reported data for cilicicone b. Therefore, compound **3** was named and assigned a stereochemistry of (*R*)-α, (*R*)-β-Dihydroxy-2′,3,4,4′,6′-pentahydroxydihydrochalcone (salsolol C) (Figure 1).

Compound **4** was purified as an amorphous pale-yellow powder, [α]^25^_D_ −10.6 (c 0.1, MeOH) and showed similar UV absorptions as those for compounds 1–3. The molecular formula was established as C_15_H_13_O_6_ by HR-DART-MS positive mode, which showed a quasi-molecular ion [M + H−H_2_O]^+^ at 289.0712 (Calcd for C_15_H_13_O_6_, 289.0713). MS and NMR data of compound **4** matched with those reported for balanochalcone, recently purified from *Balanophora laxiflora* Hemsl. (Balanophoraceae) [15]. However, the stereochemistry of the β carbon of balanochalcone was not previously assigned. Herein, we report the CD spectrum of **4** ([*θ*]_328_ +1882, [*θ*]_298_ −2096] (Supplementary Materials). The CD spectrum of **4** was similar to those recorded for **1** and **2**. Thus, the stereochemistry at the β carbon for balanochalcone was assigned with an (*S*) configuration for the first time. The systematic name of compound **4** was assigned as (*S*)-β-Hydroxy-2′,3,4′,5,6′ pentahydroxydihydrochalcone. This finding is the second time that balanochalcone has been isolated from a natural source, and it is the first report of its occurrence in the Asteraceae family.

**Figure 2:**
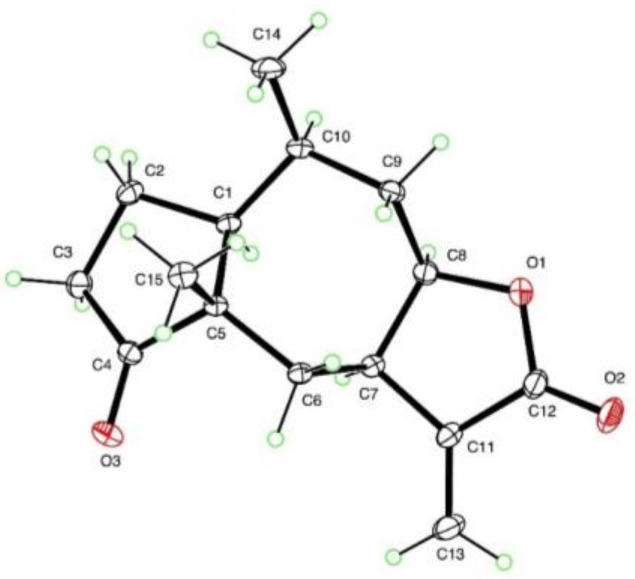
X-ray ellipsoid drawing of compound **11**, generated using the software Mercury.

Compounds **5**–**12** were unambiguously elucidated using HR-DART-MS positive mode, 1D and 2D NMR, and by comparing spectroscopic and spectrometric data with those reported in literature. The compounds were identified as follows: quercetin 3-methylether (**5**); quercetin 3,4′ dimethylether (**6**); quercetin 3,7 dimethylether (**7**), rhamnetin (**8**), quercetin (**9**), quercetin 3,4′,7 trimethylether (**10**), confertin (**11**), and neoambrosin (**12**). Additionally, high quality crystals were obtained for confertin, which allowed X-ray structural confirmation (Figure 2). Quercetin methyl derivatives are a common subclass of flavonoids found in different plant families, including Asteraceae, but as far as we know this is the first report on the purification of those flavonols from *A. salsola* [19,20]. Additionally, neoambrosin was previously isolated from *A. salsola* collected in California, USA and also from *A. monogyra* [13,21]. However, this is the first report of isolation of confertin from *A. salsola*.

### 2.2. HPLC Profile of A. salsola and A. dumosa

Chromatographic profiles of the ethyl acetate extract from *A. salsola* leaves and twigs collected from different locations and years, together with the ethyl acetate extract of *A. dumosa* collected in Arizona, were compared by HPLC (Supplementary Materials, Figure S39).

Retention times of the isolated compounds from *A. salsola* collected in Texas were assigned in the chromatogram of EtOAc crude extract (Supplementary Materials, Figure S39) and were also monitored in *A. salsola* collected from Arizona. Compounds **1–4** and **6–7** were identified by comparing their retention times and UV spectra (Supplementary Materials, Figure S39), while the remaining compounds were not detected. On the other hand, a different profile was observed for *A. dumosa*, only detecting compounds **1**, **2,** and **7**.

### 2.3. Phytotoxic and Antifungal Activity of Pure Compounds

Four of the isolated compounds were phytotoxic. Neoambrosin was only active against *A. stolonifera* at 1000 µM, while confertin was active against *L. sativa* at 1000 µM and *A. stolonifera* at 100 µM (Table 2).

**Figure 3:**
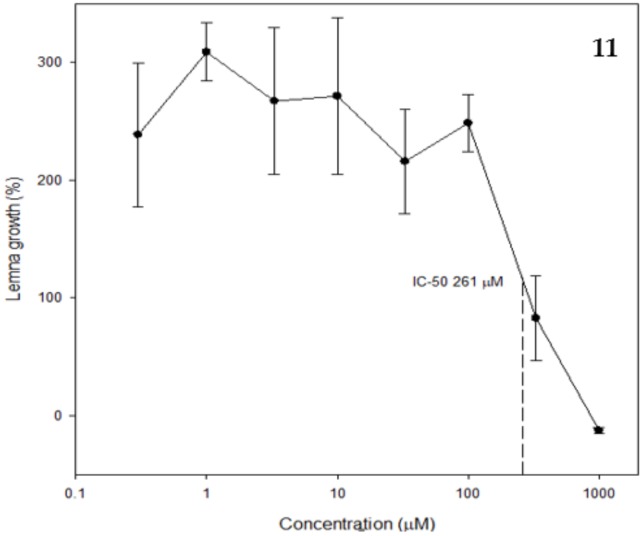
IC_50_ plot for confertin against *Lemna paucicostata* after seven days.

Confertin had an IC_50_ of 261 µM against *Lemna paucicostata*, whereas neoambrosin was not phytotoxic in this assay (Figure 3). Although, these two sesquiterpene lactones are structurally similar, the lack of a double bond in positions 1 and 2 in confertin and, in our opinion even more relevant, the five-membered lactone ring fused at positions 7 and 8 (rather than at positions 6 and 7) apparently contributed to the better phytotoxicity found in confertin than in neoambrosin. Sesquiterpene lactones have previously been reported to be phytotoxic [7,22,23,24].

Moreover, salsolols A and B were only moderately phytotoxic against *L. paucicostata*, with IC_50_ values of 275 and 251 µM (plots not shown), respectively, while the other two structurally similar dihydrochalcones had no activity. The substitution of one of the hydroxyl group of the B ring in balanochalcone for a methoxy group at position 3 (salsolol B) or 5 (salsolol A) increased the phytotoxicity remarkably. Therefore, confertin, neaombrosin, and salsolols A and B could be used as templates for the synthesis of more potent phytotoxic and fungitoxic compounds.

IC_50_ values of bioactive compounds are comparable to those of the synthetic herbicides glyphosate, asulam, and clomazone (388, 407, and 126 µM, respectively) in the same bioassay [25]. It is interesting that the most fundamental chalcone, *trans*-chalcone, is moderately phytotoxic [26,27,28]. This intermediate of the phenylpropanoid pathway may have a unique mode of action.

**Figure 4:**
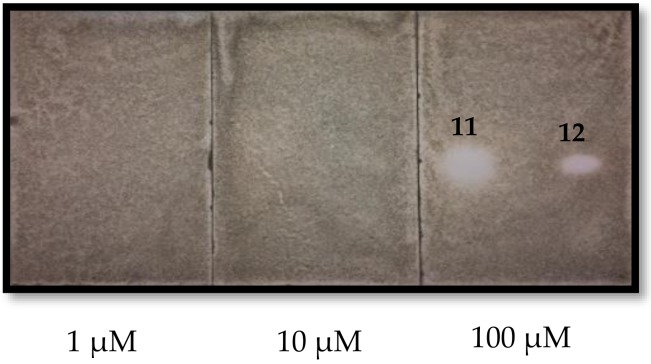
Thin layer chromatography bioautography antifungal results for confertin (**11**) and neoambrosin (**12**) at varying concentrations after spraying with spore suspensions of *Colletotrichum fragariae*.

Purified compounds were also tested against the fungal plant pathogen *Colletotrichum fragariae* using a thin layer chromatography autobiography assay. Only the pseudoguaianolide sesquiterpene lactones were antifungal at 100 µM (Figure 4). Confertin had a larger TLC inhibitory zone than neoambrosin. There was no discernible activity at 1 or 10 µM.

In summary, we described three new hydroxydihydrochalcones (salsolols A-C), two of which had moderate herbicidal activity. Confertin was also moderately herbicidal. Only the two pseudoguaianolide sesquiterpene lactones, confertin and neoambrosin, were significantly fungicidal. The bioassays done with these compounds were of limited scope, so more extensive bioassays could reveal more potent biological activities in one or more of the compounds elucidated in this study.

## 3. Materials and Methods

### 3.1. General Experimental Procedure

Fractionation of extracts and some purifications were performed using a Biotage flash chromatography system (Charlotte, NC, USA) equipped with an Isolera quaternary pump and a diode array detector set at 254 and 280 nm, using both Biotage® SNAP KP-Sil and Biotage® SNAP Ultra HP-Sphere^TM^ 25 µm silica gel flash cartridges of different sizes and a fraction collector. The final purification step was performed on a Phenomenex Luna C18(2) column (250 × 21.2 mm; 10 µm) (Charlotte, NC, USA) using a preparative HPLC (Agilent 1200 Series) equipped with a G1361A binary pump, a G2260A autosampler, a G1315A diode array detector, and a G1364B fraction collector (Santa Clara, CA, USA).

Fractions were analyzed by HPLC or on thin layer chromatography plates (250 µm silica gel plates GF with fluorescent indicator, (Analtech, Newark, DE, USA)) and visualized under UV light (at 254 and 365 nm). Fractions were sprayed with anisaldehyde spray reagent or were exposed to I_2_ vapor.

Optical rotations were determined on an Autopol IV Automatic Polarimeter model 589-546 (Rudolph Research Analytical, Hackettstown, NJ, USA). Circular dichroism (CD) spectra were recorded in an AVIV Biomedical, Inc. Model 410 SF spectrophotometer (Lakewood, NJ, USA). Direct analysis in real time-high resolution mass spectrometry analysis (DART-HRMS) of purified compounds in MeOH were acquired in an AccuTOF-DART mass spectrometer (JEOL USA, Inc. Peabody, MA, USA).

1D and 2D NMR spectra were recorded on a Bruker NMR spectrometer (Billerica, MA, USA) operating at 400 MHz (^1^H) and 100 MHz (^13^C) at 25 °C. Samples were run in MeOH-*d*_4_, CHCl_3_-*d*, or DMSO-*d*_6_. The chemical shifts were reported in *δ* (ppm) and were referenced to residual solvent signals.

### 3.2. Plant Material and Extraction

*Ambrosia salsola* species were collected in two locations: in Cameron county, Texas (2006) and near Ivins, Utah (N 37.1727694° W 113.7118583°, USDA ARS Poisonous Plant Research Laboratory Herbarium, PPRL 4755) in May 2018. *A. dumosa* was collected near Beaver Dam, Arizona (N 36.9715278° W 113.8740555°, USDA ARS Poisonous Plant Research Laboratory Herbarium, PPRL 4756) in May 2018. Twigs and leaves (373 g) collected in Texas were ground into powder and successively extracted three times (1.5 L × 3) with hexane, ethyl acetate, and methanol at room temperature to afford hexane extract (4.5 g), ethyl acetate extract (24 g), and methanol extract (24.2 g). Twig and leaf material collected in Arizona and Utah were air dried at ambient temperature, and subsequently extracted using the same method as described for the Texas collection.

### 3.3. Isolation Procedure

Ethyl acetate extract (24 g) of *A. salsola* collected in Texas was dissolved in ethyl acetate and absorbed onto a silica samplet (i.d. 7 cm), and was fractionated in a silica gel 340 g cartridge using Biotage flash chromatography. Gradient elution was obtained with a mixture of hexane:ethyl acetate (100:0 to 0:100) and ethyl acetate:acetone (100:0 to 70:30) to afford 14 fractions: 1 (212 mg); 2 (204 mg); 3 (113 mg); 4 (178 mg); 5 (292 mg); 6 (850 mg); 7 (383 mg); 8 (1.16 g); 9 (1.54 g); 10 (5.5 g); 11 (6.5 g); 12 (2.4 g); 13 (1.3 g); and 14 (2.74 g). Fraction 11 (6.5 g) was subjected to flash chromatography using a 100 g silica gel cartridge and a gradient elution with hexane:ethyl acetate (70:30 to 0:100) to afford 11 fractions: 1.1 (18 mg); 1.2 (17 mg); 1.3 (272 mg); 1.4; 151 mg); 1.5 (150 mg); 1.6 (247 mg); 1.7 (470 mg); 1.8 (758 mg); 1.9 (1.72 g); 1.10 (637 mg); and 1.11 (891 mg). Fraction 1.3 (272 mg) was run in a preparative RP-C18 HPLC (250 × 21.2 mm; 10 µm) using acetonitrile:0.1% formic acid in water (35:65 *v*/*v*) at 12 mL/min to yield compound **1** (7.9 mg). Fractions 8 (1.16 g) and 9 (1.5 g) were combined and run in 100 g silica gel cartridge using dichloromethane:ethyl acetate (100:0 to 60:40) to give 13 fractions: 2.1 (672 mg); 2.2 (504 mg); 2.3 (122 mg); 2.4 (33 mg); 2.5 (47 mg); 2.6 (30 mg); 2.7 (30 mg); 2.8 (25 mg); 2.9 (25 mg); 2.10 (120 mg); 2.11 (86 mg); 2.12 (80 mg); and 2.13 (76 mg). Fraction 2.3 was run in a preparative RP-C18 HPLC using acetonitrile:0.1% formic acid in water (35:65 *v*/*v*) to yield compound **2** (7.7 mg). Fraction 10 (5.5 g) was chromatographed using a 340 g silica gel cartridge and run with hexane:ethyl acetate (100:0 to 0:100) to afford 12 fractions: 3.1 (101 mg); 3.2 (188 mg); 3.3 (1.39 g); 3.4 (302 mg); 3.5 (354 mg); 3.6 (303 mg); 3.7 (519 mg); 3.8 (758 mg); 3.9 (852 mg); 3.10 (459 mg); 3.11 (119 mg); and 3.12 (140 mg). Fractions 3.9 to 3.12 were chromatographed in a 100 g silica gel cartridge with hexane:ethyl acetate (90:10 to 60:40) to afford compound **3** (173 mg) and compound **5** (49 mg). Fractions 3.4 to 3.6 were combined and run in RP C-18 chromatography using a Phenomenex Luna C18(2) column (250 × 21.2 mm; 10 µm) with acetonitrile:0.1% formic acid in water (40:60 *v*/*v*) to afford compounds **4** (88 mg), **6** (10 mg), and **7** (12 mg). Compounds **8** (39 mg) and **9** (61 mg) were precipitated from fractions 9 and 1.8, respectively. From fraction 3.3, an additional 60 mg of compound **8** was precipitated and the supernatant was run in a 50 g silica cartridge with dichloromethane: ethyl acetate to give compound **10** (10 mg). Fraction **2.1** was crystallized in dichloromethane:ethyl acetate (8:2) to give compound **11** (326 mg), and the supernatant was run in an RP C-18 preparative HPLC column with acetonitrile:0.1% formic acid in water (60:40, v/v) to get additional compound **12** (90 mg) (Isolation flow chart in Supplementary Materials).

### 3.4. Physical Chemical Data

*Salsolol A* (**1**): Amorphous yellowish powder; [α]^25^_D_ −20.0 (c 0.1, MeOH), RP-C18 HPLC retention time 13.1 min; HR-DART-MS (positive mode) *m*/*z* 303.0920 [M + H−H_2_O]^+^ (Calcd for C_16_H_15_O_6_, 303.0869); ^1^H and ^13^C NMR chemical shift assignments are shown in Table 1.

*Salsolol B* (**2**): Amorphous pale-yellow powder; [α]^25^_D_ −12.1 (c 0.1, MeOH), RP-C18 HPLC retention time 12.8 min; HR-DART-MS (positive mode) *m*/*z* 303.0910 [M + H−H_2_O]^+^ (Calcd for C_16_H_15_O_6_, 303.0869); ^1^H and ^13^C NMR chemical shift assignments are shown in Table 1.

*Salsolol C* (**3**): Amorphous brownish powder; [α]^25^_D_ +10.0 (c 0.1, MeOH), RP-C18 HPLC retention time 6.8 min; HR-DART-MS (positive mode) *m*/*z* 305.0650 [M + H−H_2_O]^+^ (Calcd for C_15_H_13_O_7_, 305.0662); ^1^H and ^13^C NMR chemical shift assignments are shown in Table 1.

*Balanochalcone* (**4**): Amorphous pale yellow powder, [α]^25^_D_ −10.6 (c 0.1, MeOH), RP-C18 HPLC retention time 10.3 min; HR-DART-MS (positive mode) *m*/*z* 289.0748 [M + H−H_2_O]^+^ (calcd for C_15_H_13_O_6_, 289.0713); ^1^H and ^13^C NMR; δ_H_/δ_C_ (MeOH-*d*_4_): 131.9 (qC-1); 6.91/114.9 (H-2/C-2); 147.0 (C-3); 6.79/116.4 (H-4/C-4); 146.7 (C-5); 6.79/116.4 (H-6/C-6); 5.27/80.6 (H*β*/C*β*); 2.68; 3.03/44.2 (H*α*/C*α*); 197.9 (C*β*′*)* 103.5 (qC-1′); 165.0 (qC-2′); 5.91/96.3 (H-3′/C-3′); 168.5 (qC-4′); 5.91/97.2 (H-5′/C-5′); 165.6 (H-6′/C-6′).

### 3.5. HPLC Analysis

Ethyl acetate extracts from *A. salsola* and *A. dumosa* (10 mg/mL) and pure compounds (1 mg/mL) were analyzed in a 1260 Agilent HPLC. 5 µL of the samples were injected in a Phenomenex Luna C18(2) column (250 × 4.6 mm; 10 µm) using a gradient mode elution with formic acid (0.1%) in water (**A**) and acetonitrile (**B**) as follows: 0–2 min, 25% B; 2–15 min, 25–70% B; 15–21 min, 70–95% B; washed with 95% B over 2 min and equilibrated with 25% B over 4 min. The flow rate was set at 1 mL/min. The wavelengths were set at 254 and 280 nm and the column temperature at 40 °C.

### 3.6. X-ray Crystallography of Compound 11

The crystal structure of confertin (**11**) was determined from a colorless crystal of dimensions 0.42 × 0.28 × 0.24 mm, using data collected at T = 90 K with Mo Kα radiation on a Bruker APEX-II DUO CCD diffractometer, equipped with an Oxford Cryostream cooler. The structure was solved using the program SHELXS-97 and refined anisotropically by full-matrix least-squares on F^2^ using SHELXL-2014/7 [29]. All 20 H atoms were visible in difference maps but were placed in idealized positions for the refinement.

Crystal data: C_15_H_20_O_3_ Mr = 248.31, monoclinic space group P2_1_, a = 7.1249(6) Å, b = 10.1761(9) Å, c = 9.5681(8) Å, β = 109.597(4)°, V = 653.54(10) Å^3^, Z = 2, Dx = 1.262 Mg m^−3^, θ_max_ = 50.7°, 42413 measured data, 13792 unique, R_int_ = 0.047. R = 0.032 for 12740 data with I > 2*σ* (I) and 0.036 for all unique data and 165 refined parameters. The Flack parameter is 0.04(16) for 5648 Bijvoet quotients, in agreement with the accepted absolute configuration of sesquiterpene lactones from higher plants [30]. Supplementary crystallographic data for **11** are contained in Cambridge Structural Database deposition CCDC-1868301; this data can be obtained free of charge via www.ccdc.cam.ac.uk/conts/retrieving.html (or from the Cambridge Crystallographic Data Centre, 12 Union Road, Cambridge CB2 1EZ, UK; fax: +44-1223-336-033; or e-mail: deposit@ccdc.cam.ac.uk).

### 3.7. Phytotoxicity Bioassay

#### 3.7.1. *Lactuca sativa* and *Agrostis stolonifera*

Crude extracts, fractions, and pure compounds were evaluated for germination and growth inhibition of dicotyledon and monocotyledon *Lactuca sativa* L. (lettuce) and *Agrostis stolonifera* L. (bentgrass), respectively, in 24-well plates using a previously described method [31]. Plates were incubated in a Percival Scientific CU-36 L5 incubator under 16:8 h light/dark conditions at 26 °C and 120 μmol s^−1^ m^−2^ average photosynthetically active photon flux. After 7 days for *L. sativa* and 10 days for *A. stolonifera*, seed germination and growth were compared with a positive control (solvent well plants) and ranked from 0 to 5. A ranking of 0 indicated that sample well plants grew the same amount as the control. A ranking of 5 indicated no germination.

#### 3.7.2. *Lemna paucicostata*

Compounds were also evaluated using a *Lemna paucicostata* (duckweed) microbioassay to determine IC_50_ growth effects [31]. In this assay, two duckweed plants with two three-frond colonies from 4- to 5-day-old stock cultures were placed in each well of a six-well plate with 4950 µL of modified Hoagland’s media and 50 µL of water, or solvent, or compound dissolved in the appropriate solvent. The final concentration of the solvent (acetone or EtOH) was 1% by volume. Modified Hoagland media contained 1515 mg/L KNO_3_, 680 mg/L KH_2_PO4, 492 mg/L MgSO_4_ × 7 H_2_O, 20 mg/L Na_2_CO_3_, 1180 mg/L Ca(NO_3_)_2_ × 4 H_2_O, 0.5 mg/L MnCl_2_, 0.025 mg/L CoCl_2_, 0.025 mg/L CuSO_4_ × 5 H_2_O, and 18.355 mg/L Fe-EDTA. The media was adjusted to pH 5.5 with 1 M NaOH and filtered through a 0.2 µm filter. All six-well plates were incubated in the Percival incubator as described above. Plant frond areas were measured at day 0 and day 7 using a Lemnatec Scanalyzer PL with LemnaLauncher and LemnaMiner software version 2.1.0.8646 (LemnaTec GmbH, Würselen, Germany). This image analysis software was used to measure and monitor frond number, total frond area, as well as color classes (healthy, chlorotic, and necrotic tissue). Replicate tests at varying concentrations of test compounds allowed for determination of IC_50_ values using R Studio software (v. 0.99.491).

### 3.8. Antifungal Bioautography Assay

The evaluation of the antifungal activity against fungal plant pathogens was performed using a published TLC bioautography procedure [32]. The sensitivity of fungal species to each tested compound was determined by comparing the sizes of the inhibitory zones. Bioautography experiments were performed multiple times using both dose- and non-dose-response. Fungicide technical grade standards benomyl (98%), cyprodinil (98%), azoxystrobin (98%), and captan (98%; Chem Service, Inc., West Chester, PA, USA) were used as controls. Pure compounds (10 µL) were spotted at 1, 10, and 100 µM on silica gel TLC plates (250 μm, silica gel GF Uniplate; Analtech, Inc., Newark, DE, USA) that were sprayed with spore suspensions of *Colletotrichum fragariae* adjusted to a final concentration of 3.0 × 10^5^ conidia/mL with liquid potato dextrose broth (PDB, Difco) and 0.1% Tween-80. Using a 50 mL chromatographic sprayer, each TLC plate was sprayed lightly (to a damp appearance) three times with the conidial suspension. Inoculated plates were then placed in a 30 × 13 × 7.5 cm transparent moisture chamber (39 °C, 100% relative humidity; Pioneer Plastics, Inc.) and incubated over four days in a growth chamber at 24 ± 1°C and a 12 h photoperiod under 60 ± 5 μmols·m^−2^ s^−1^ light fungal growth. Inhibition was determined by comparing the size of the inhibitory zones on the TLC plate.

## Figures and Tables

**Table 1:** ^1^H and ^13^C NMR spectroscopic data of compounds **1**–**3**.

Position	1 *^a^*		2 *^a^*		3 *^a^*	
	δ_H (ppm)_	δ_C (ppm)_	δ_H (ppm)_	δ_C (ppm)_	δ_H (ppm)_	δ_C (ppm)_
1	-	133.3	-	132.0	-	130.0
2	6.95 d (*J* = 1.9 Hz, 1H)	114.7	7.07 d (*J* = 2.0 Hz, 1H)	111.5	6.97 d (*J* = 1.9 Hz, 1H)	116.2
3	-	147.9	-	149.3	-	146.5
4	6.91 d, 1H	112.8	-	148.3	-	147.3
5	-	149.5	6.82 d (*J* = 8.1 Hz, 1H)	116.3	6.80 d (*J* = 8.1 Hz, 1H)	116.0
6	6.91 d, 1H	119.1	6.92 dd (*J* = 8.1, 2.0 Hz, 1H)	120.7	6.85 dd (*J* = 8.1, 1.9 Hz, 1H)	121.0
*β*	5.31 dd (*J* = 12.6, 3.0 Hz, 1H)	80.4	5.34 dd (*J* = 12.8, 3.0 Hz, 1H)	80.8	4.91 d (*J* = 11.4 Hz, 1H)	85.3
*α*	2.71 dd (*J* = 17.1, 3.0 Hz,1H) 3.05 dd (*J* = 17.1, 12.6 Hz,1H)	44.2	2.70 dd (*J* = 17.1, 3.0 Hz,1H) 3.12 dd (*J* = 17.1, 12.8 Hz,1H)	44.3	4.50 d (*J* = 11.4 Hz,1H)	73.8
*β*′	-	197.7	-	197.6	-	198.5
1′	-	103.5	-	103.2	-	102.0
2′	-	164.9	-	165.0	-	164.6
3′	5.89 d (*J* = 2.2 Hz, 1H)	97.3	5.89 d (*J* = 2.2 Hz, 1H)	97.6	5.92 d (*J* = 2.0 Hz, 1H)	97.5
4′	-	168.7	-	169.8	-	168.8
5′	5.87 d (*J* = 2.2 Hz, 1H)	96.4	5.87 d (*J* = 2.2 Hz, 1H)	96.1	5.89 d (*J* = 2.0 Hz, 1H)	96.4
6′	-	165.6	-	165.7	-	165.4
3-OCH_3_	-	-	3.88 s (3H)	56.7	-	-
5-OCH_3_	3.86 s (3H)	56.6	-	-		

*^a^* NMR data were recorded in MeOH-*d*_4_.

**Table 2:** Phytotoxicity of active purified compounds from *A. salsola*.

Compounds	C (µM)	*L. sativa*	*A. stolonifera*
**Confertin**	10	0	0
	100	0	3
	1000	4	5
**Neoambrosin**	10	0	0
	100	0	0
	1000	0	4

Ranking based on scale of 0 to 5. 0 = no effect. 5 = no growth.

## Supplementary Materials

The following are available online, Figure S1–S39: UV, Circular Dichroism, HPLC chromatogram of ethyl acetate extracts of *Ambrosia salsola* and *Ambrosia dumosa*, HR-DART-MS, 1D and 2D NMR spectra of salsolol A (1), salsolol B (2), salsololo C (3) and balanochalcone (4).
