# Supplementary material for: Bioassay-Guided Isolation and Structure Elucidation of Fungicidal and Herbicidal Compounds from Ambrosia salsola (Asteraceae)
Source: Molecules. 2019 Feb 26;24(5):835. doi: 10.3390/molecules24050835 (PMC6429092; doi:10.3390/molecules24050835)
Supplement: Supplementary file 1 [file molecules-24-00835-s001.pdf]

SUPPORTING INFORMATION

Bioassay-guided isolation and structure elucidation of fungicidal and herbicidal compounds from *Ambrosia salsola* (Asteraceae).

Wilmer H. Perera,[1]* Kumudini M. Meepagala,[2]* Frank R. Fronczek,[3] Daniel D. Cook,[4] David E. Wedge[4] and Stephen O. Duke[4]

[1]ORISE Fellow-Agricultural Research Service, U.S. Department of Agriculture, Natural Products Utilization Research Unit, P.O. Box 1848, University, MS, 38677, USA; wilmer.perera@gmail.com (W.H.P.)
[2]Agricultural Research Service, U.S. Department of Agriculture, Natural Products Utilization Research Unit, P.O. Box 1848, University, MS, 38677, USA; Kumudini.Meepagala@ars.usda.gov (K.M.M); david.wedge@ars.usda.gov (D.E.W); Stephen.Duke@ars.usda.gov (S.O.D)
[3]Department of Chemistry, Louisiana State University, Baton Rouge, LA 70803, USA; ffroncz@lsu.edu (F.R.F)
[4]Agricultural Research Service, U.S. Department of Agriculture, Poisonous Plant Research Lab. 1150 E 1400 N. N Logan, UT 84341, USA; Daniel.Cook@ars.usda.gov (D.D.C)

*****Correspondence: Wilmer.Perera@gmail.com; Tel: (662)915-1046 (W.H.P); Kumudini.Meepagala@ars.usda.gov; Tel: (662)915-1030 (K.M.M)

Abstract: The discovery of potent natural and ecofriendly pesticides is one of the focuses of the agrochemical industry, and plant species are a source of many potentially active compounds. We describe the bioassay-guided isolation of antifungal and phytotoxic compounds from the ethyl acetate extract of *Ambrosia salsola* twigs and leaves. With this methodology, we isolated and identified twelve compounds (four chalcones, six flavonols and two pseudoguaianolide sesquiterpene lactones). Three new chalcones were elucidated as (*S*)-β-Hydroxy-2',3,4',6' tetrahydroxy, 5 methoxydihydrochalcone (salsolol A), (*S*)-β-Hydroxy-2',4,4',6' tetrahydroxy, 3 methoxydihydrochalcone (salsolol B) and (*R*)-α, (*R*)-β-Dihydroxy-2',3,4,4',6' pentahydroxydihydrochalcone (salsolol C) together with nine known compounds: balanochalcone, six quercetin derivatives, confertin and neoambrosin. Chemical structures were determined based on comprehensive HR-DART-MS, as well as 1D and 2D NMR experiments (DQFCOSY, HMQC and HMBC), and the absolute configurations of the chalcones were confirmed by CD spectra analysis. Crystal structure of confertin was determined by X-ray diffraction. The phytotoxicity of purified compounds was evaluated, and neoambrosim was active against *Agrostis stolonifera* at 1 mM, while confertin was active against both, *Lactuca sativa* and *A. stolonifera* at 1 mM and 100 µM, respectively. Confertin and salsolol A and B had IC_{50} values of 261, 275 and 251 µM, respectively, against *Lemna pausicotata* (duckweed). The antifungal activity was also tested against *Colletotrichum fragariae* Brooks using a thin layer chromatography bioautography assay. Both, confertin and neoambrosin were antifungal at 100 µM, with confertin's activity beings higher than that of neoambrosin at this concentration.

Keywords: *Ambrosia salsola*, *Ambrosia dumosa*, β-Hydroxydihydrochalcones, fungitoxic and phytotoxic compounds, confertin, X-Ray diffraction

Figures	Pages
**Figure S1**: Isolation procedure flow chart	1
**Figure S2:** UV spectrum of compound 1	2
**Figure S3:** CD spectrum of compound 1	2
**Figure S4:** Chemical structure of compound 1	2
**Figure S5:** HR-DART-MS positive mode ion of compound 1	3
**Figure S6:** ^{1}H-NMR spectrum of compound 1	4
**Figure S7:** ^{13}C-NMR spectrum of compound 1	5
**Figure S8:** DEPT 135 spectrum of compound 1	6
**Figure S9:** COSY spectrum of compound 1	7
**Figure S10**: HMQC spectrum of compound 1	8
**Figure S11**: HMBC spectrum of compound 1	9
**Figure S12:** UV spectrum of compound 2	10
**Figure S13:** CD spectrum of compound 2	10
**Figure S14:** Chemical structure of compound 2	10
**Figure S15:** HR-DART-MS positive mode ion of compound 2	11
**Figure S16:** ^{1}H-NMR spectrum of compound 2	12
**Figure S17:** ^{13}C-NMR spectrum of compound 2	13
**Figure S18:** DEPT 135 spectrum of compound 2	14
**Figure S19:** COSY spectrum of compound 2	15

Figures	Pages
**Figure S20**: HMQC spectrum of compound 2	16
**Figure S21**: HMBC spectrum of compound 2	17
**Figure S22:** UV spectrum of compound 3	18
**Figure S23:** CD spectrum of compound 3	18
**Figure S24:** Chemical structure of compound 3	18
**Figure S25:** HR-DART-MS positive mode ion of compound 3	19
**Figure S26:** ^{1}H-NMR spectrum of compound 3	20
**Figure S27:** ^{13}C-NMR spectrum of compound 3	21
**Figure S28:** DEPT 135 spectrum of compound 3	22
**Figure S29:** COSY spectrum of compound 3	23
**Figure S30:** HMQC spectrum of compound 3	24
**Figure S31:** HMBC spectrum of compound 3	25
**Figure S32:** UV spectrum of compound 4	26
**Figure S33:** CD spectrum of compound 4	26
**Figure S34:** Chemical structure of compound 4	26
**Figure S35:** HR-DART-MS positive mode ion of compound 4	27
**Figure S36:** ^{1}H-NMR spectrum of compound 4	28
**Figure S37:** ^{13}C-NMR spectrum of compound 4	29
**Figure S38:** DEPT 135 spectrum of compound 4	30
**Figure S39:** HPLC chromatograms of ethyl acetate extracts (10 mg/mL) recorded at 254 nm for *A. salsola* collected in Texas **(A)**, *A. salsola* from Arizona **(B)** and *A. dumosa* **(C)**.	30

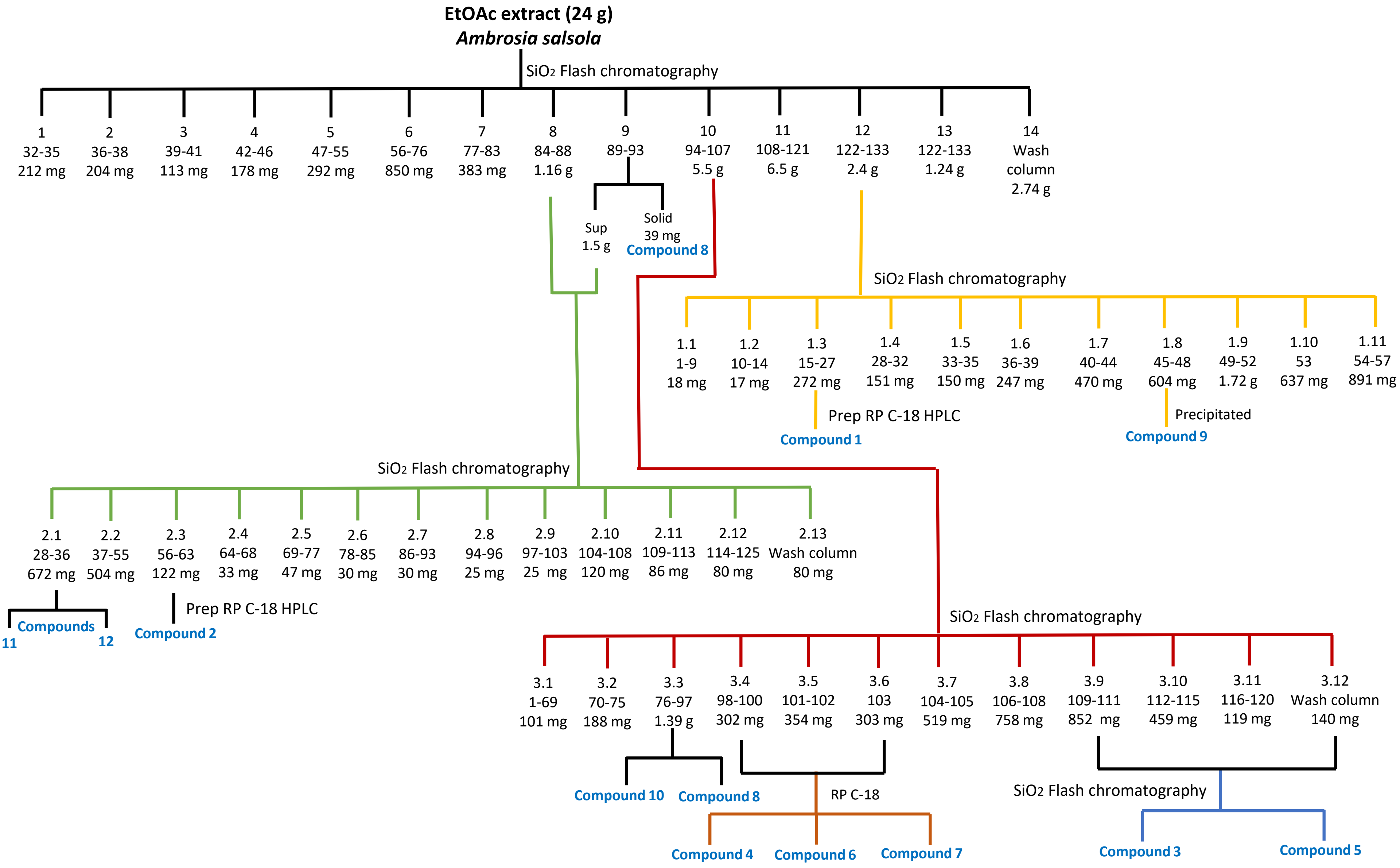

Figure S1: Isolation procedure flow chart.

Compound 1 (Salsolol A)

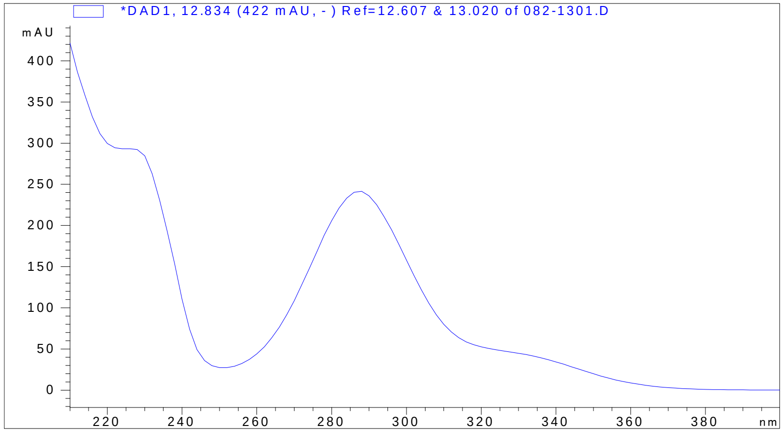

Figure S2: UV spectrum of compound 1.

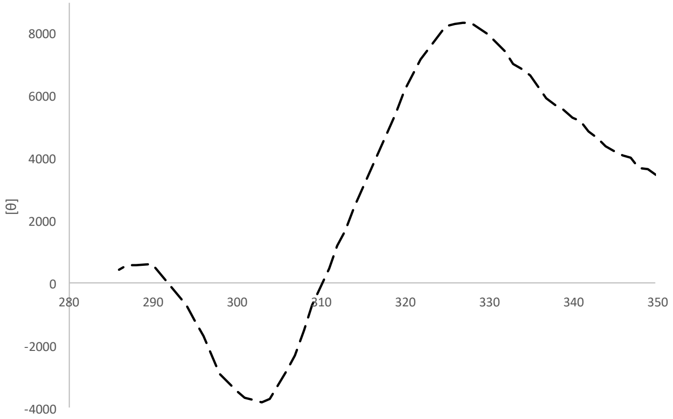

Figure S3: CD spectrum of compound 1.

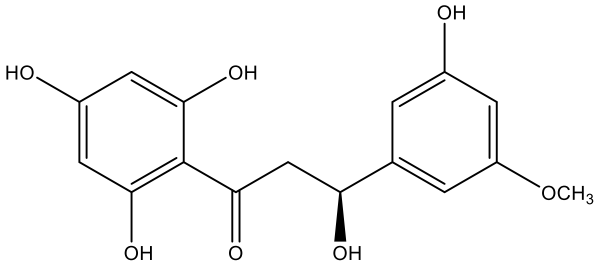

Chemical Formula: $C_{16}H_{16}O_7$
Molecular Weight: 320.30

Figure S4: Chemical structure of compound 1.

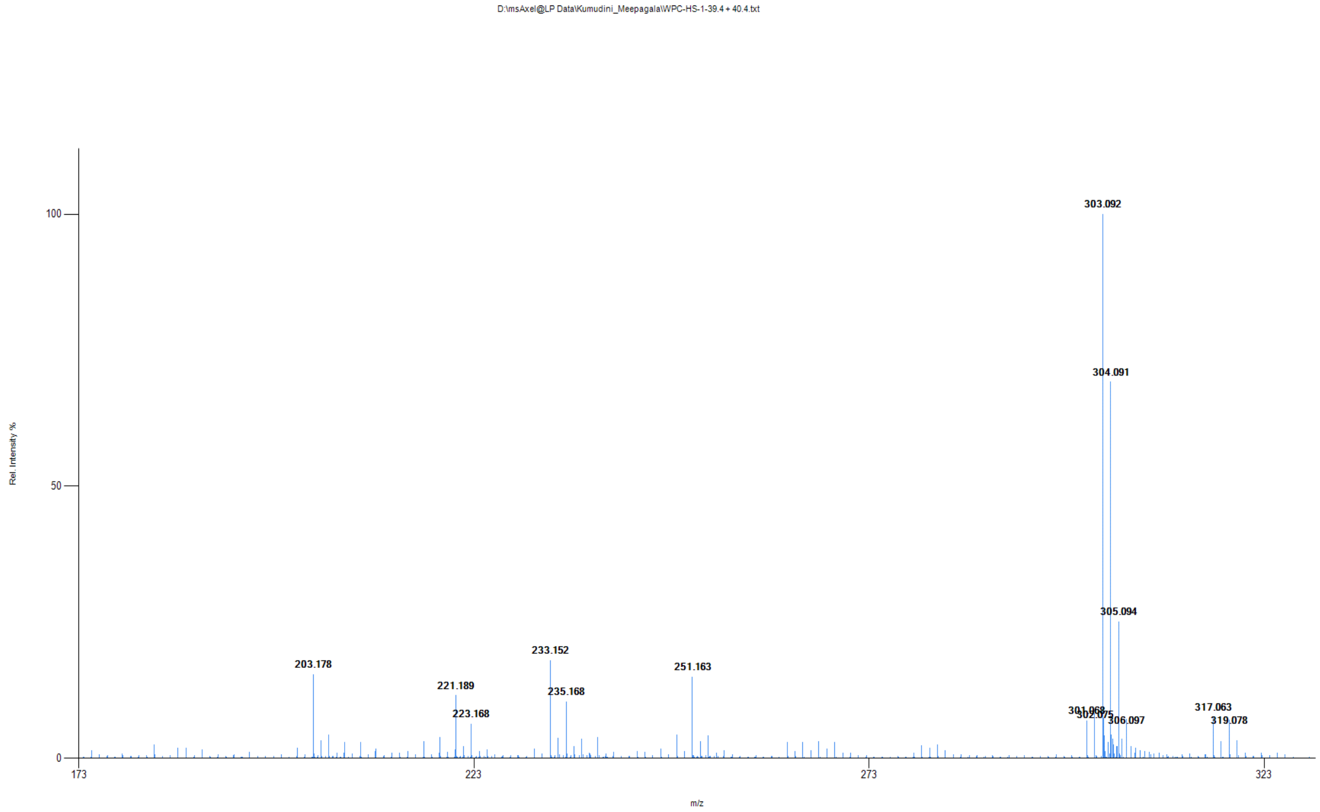

Elemental Compositions

Element Limits: C 0/50 H 0/100 O 0/10

Tolerance: 7 mmu   Even or odd electron ion or both: Both

Electron correction: None.   Charges: 1

Minimum unsaturation: -1   Maximum unsaturation: 100

Calc. m/z	Abund %	mmu	DBE	Composition
303.086863	100.000	-5.09	9.5	C16H15O6

Figure S5: HR-DART-MS positive mode ion of compound 1.

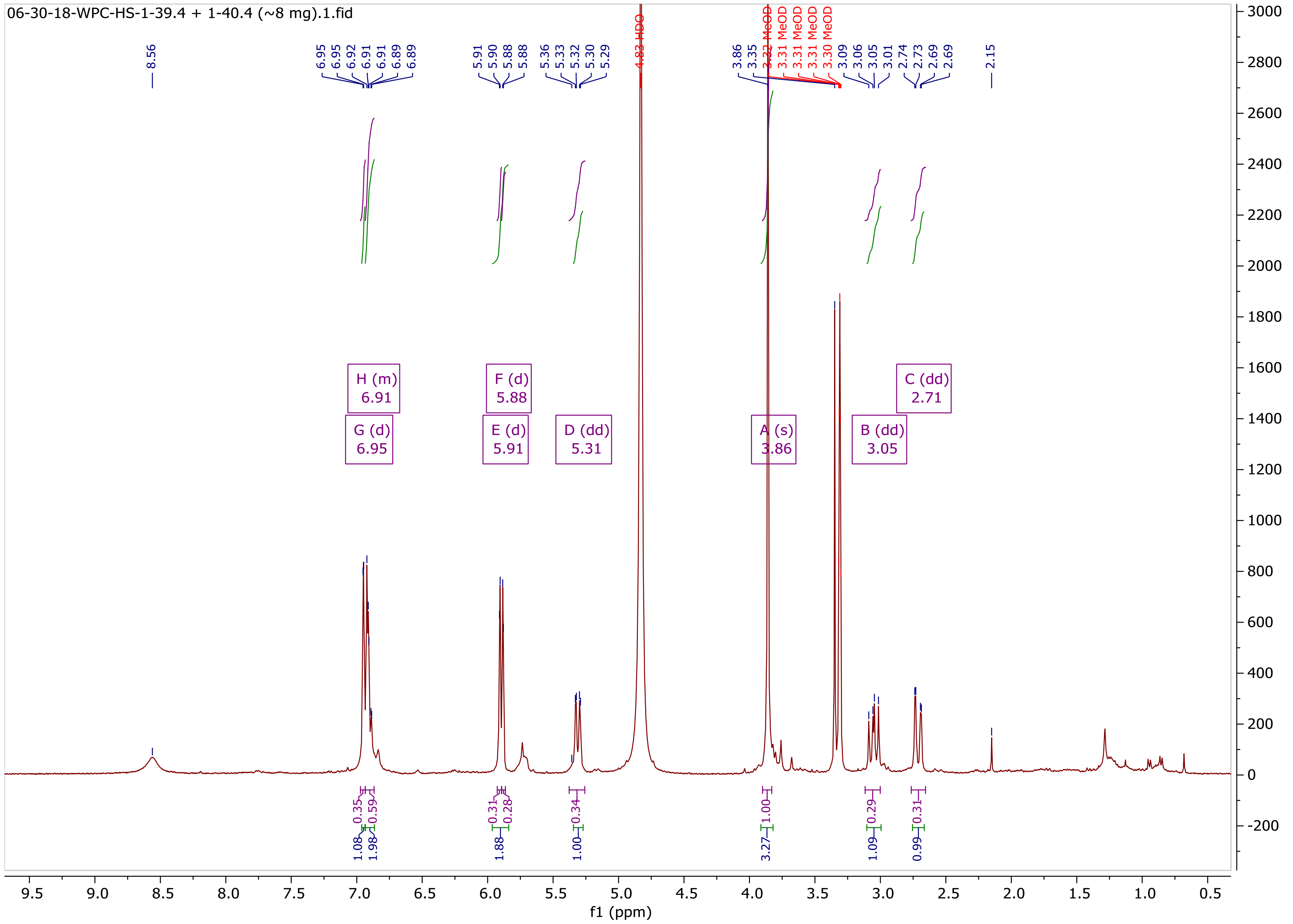

Figure S6: ^{1}H-NMR spectrum of compound 1.

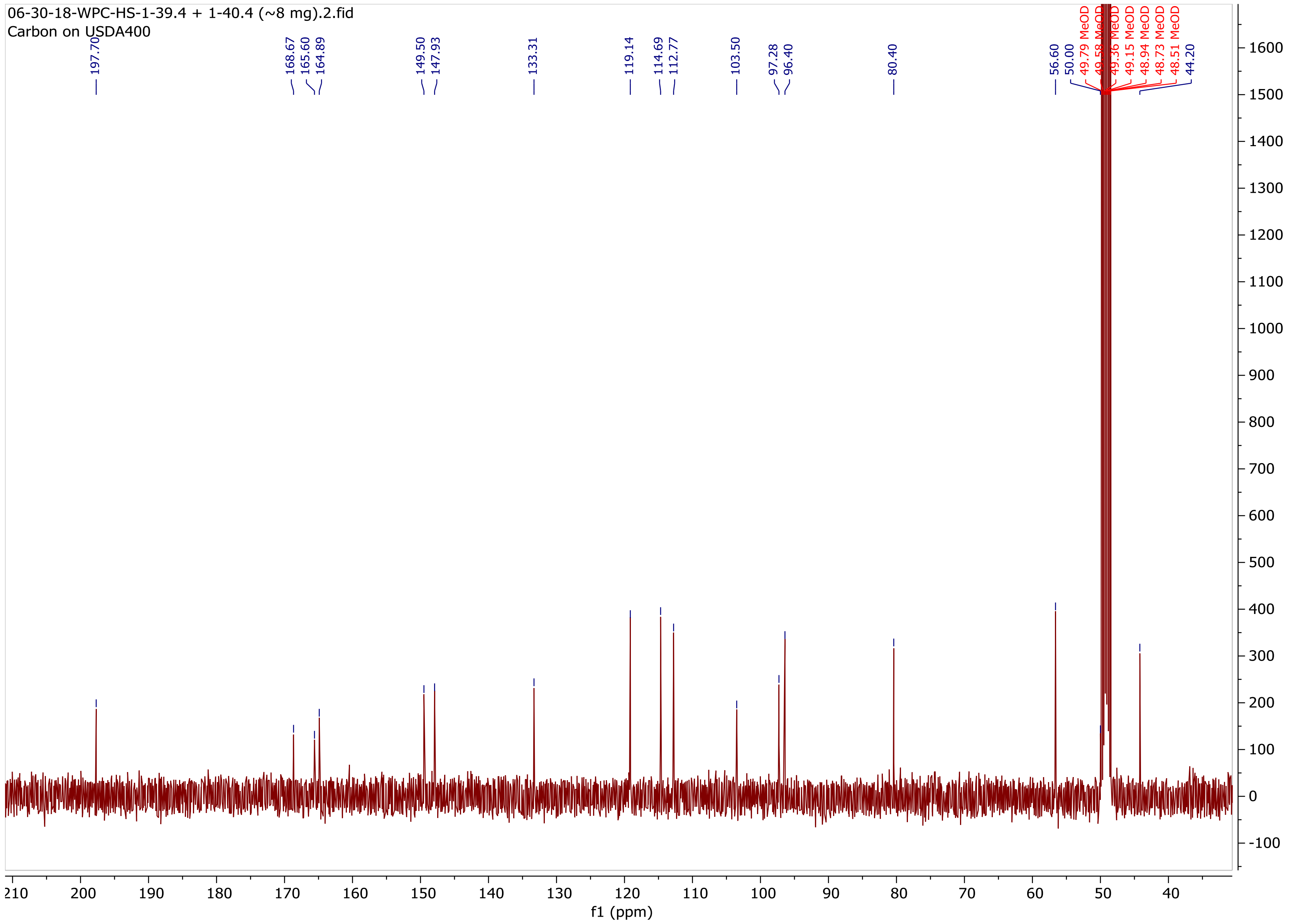

Figure S7: ^{13}C-NMR spectrum of compound 1.

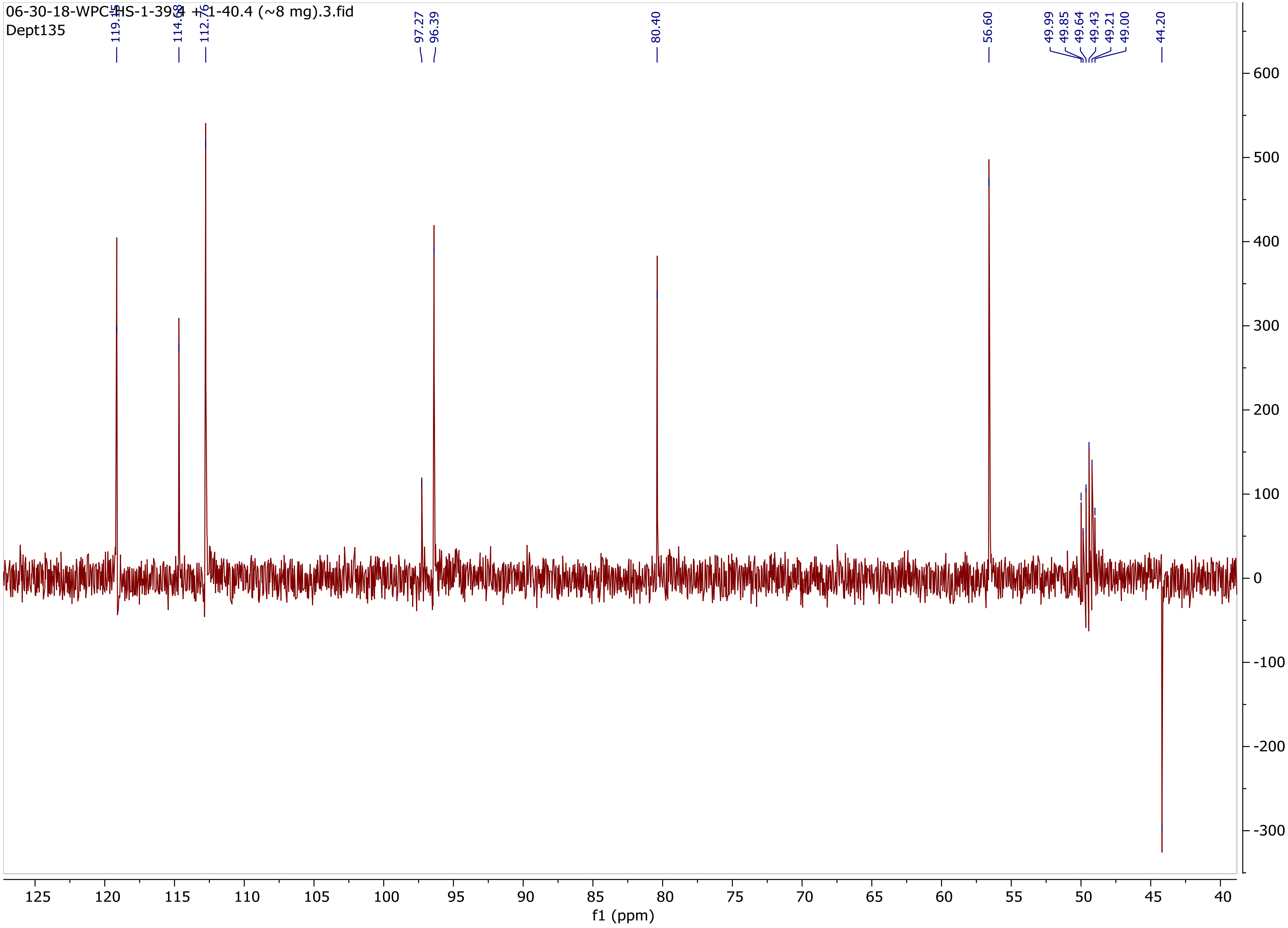

Figure S8: DEPT 135 spectrum of compound 1.

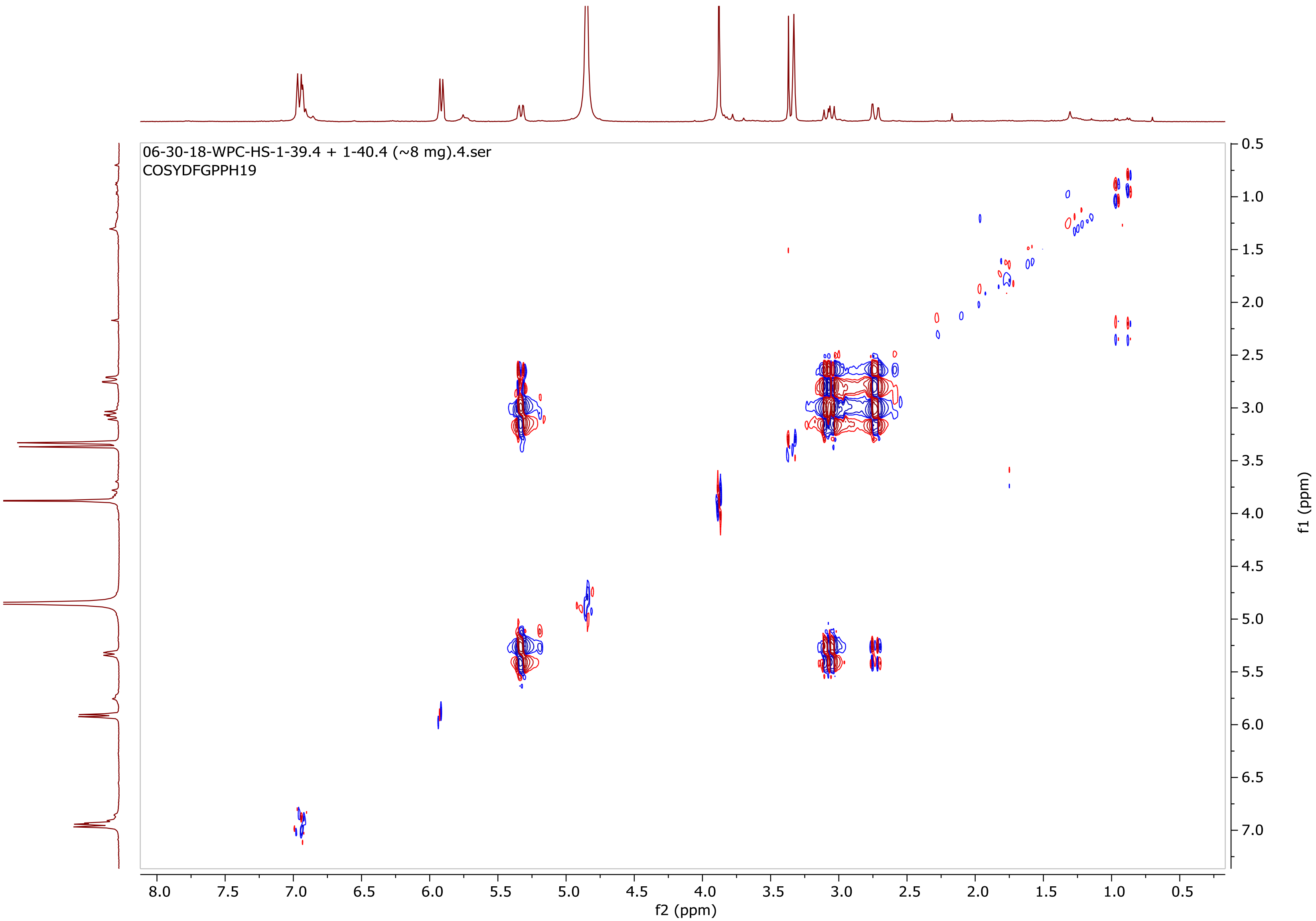

Figure S9: COSY spectrum of compound 1.

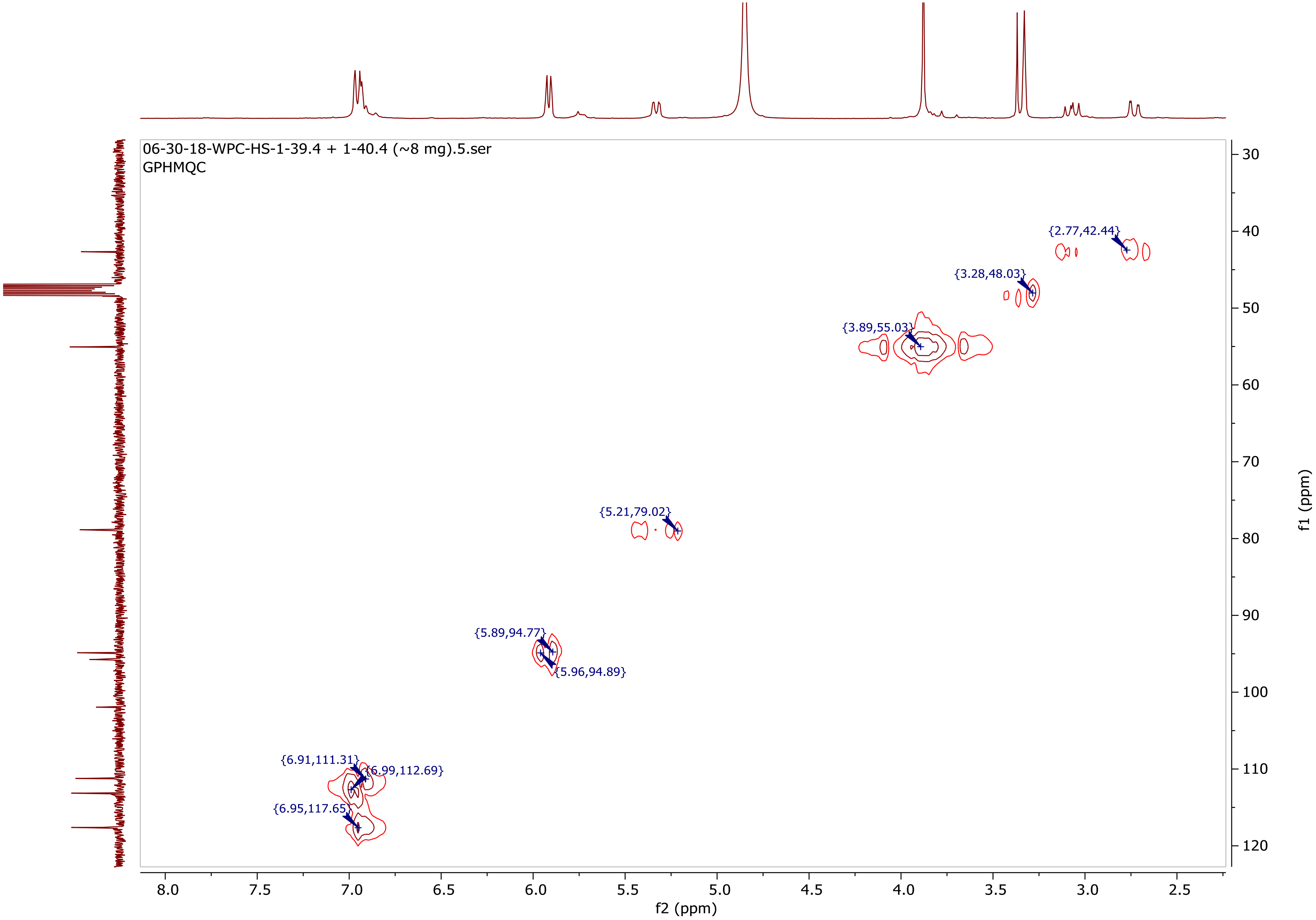

Figure S10: HMQC spectrum of compound 1.

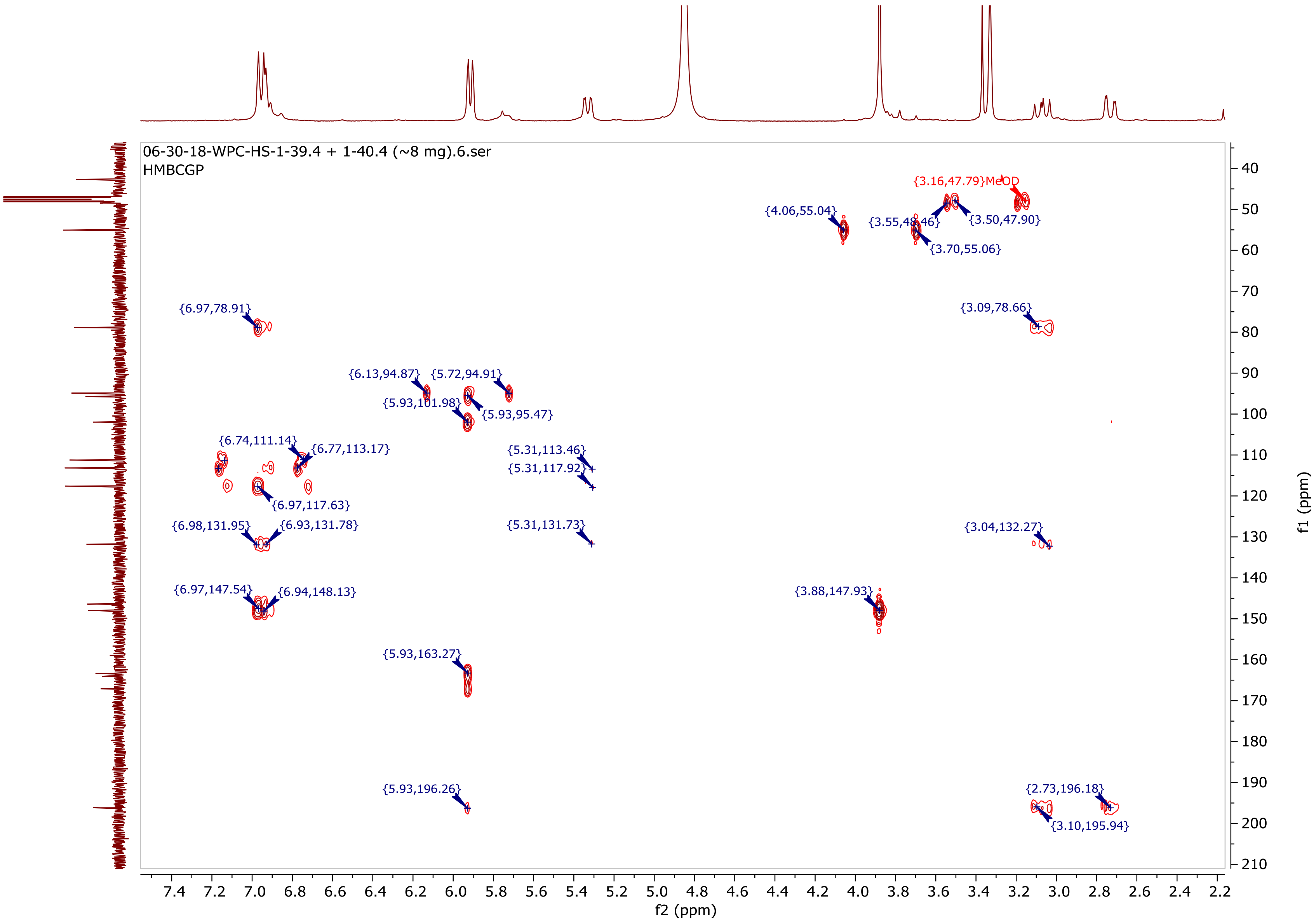

Figure S11: HMBC spectrum of compound 1.

Compound 2 (Salsolol B)

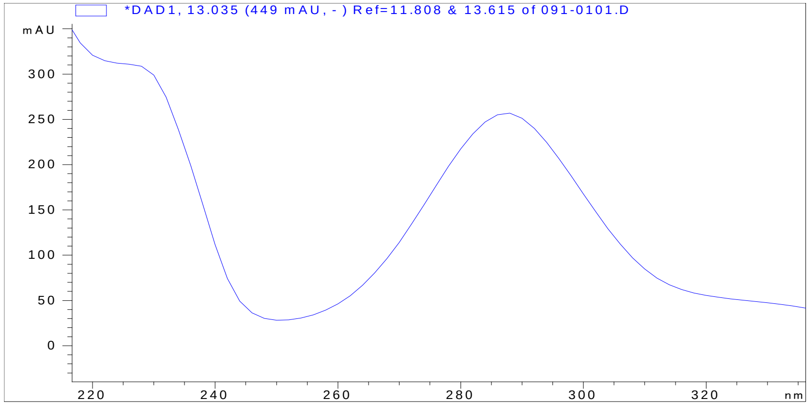

Figure S12: UV spectrum of compound 2.

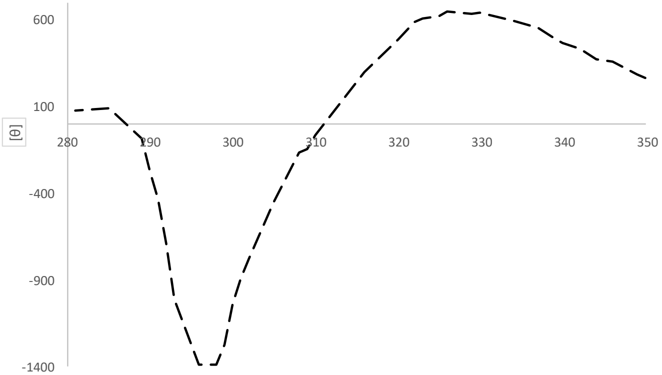

Figure S13: CD spectrum of compound 2.

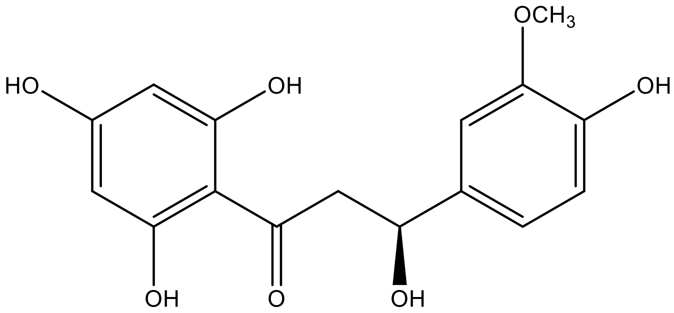

Chemical Formula: $C_{16}H_{16}O_7$

Molecular Weight: 320.30

Figure S14: Chemical structure of compound 2.

D:\msAxel@LP Data\Kumudini_Meepagala\WPC-HS-1-40.1 + 40.2.txt

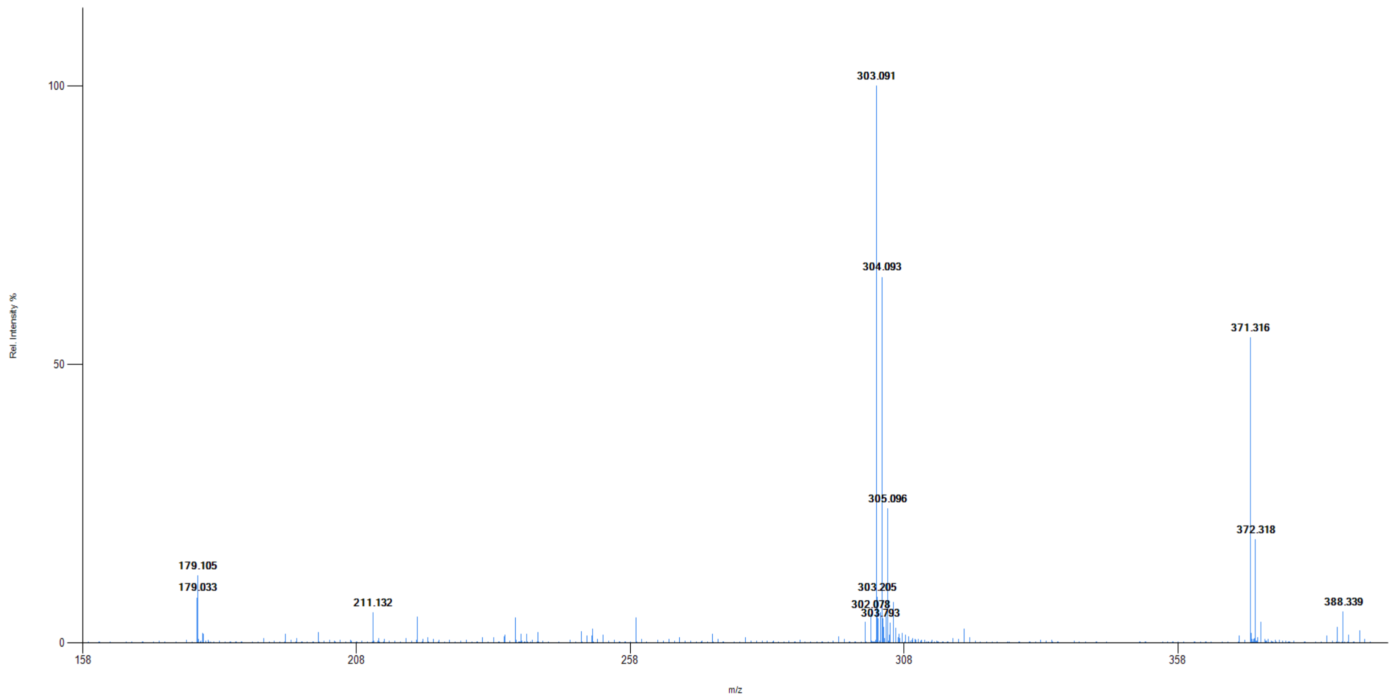

Elemental Compositions

Element Limits: C 0/50 H 0/100 O 0/10

Tolerance: 7 mmu   Even or odd electron ion or both: Both

Electron correction: None.   Charges: 1

Minimum unsaturation: -1   Maximum unsaturation: 100

Calc. m/z	Abund %	mmu	DBE	Composition
303.086863	100.000	-4.60	9.5	C16H15O6

Figure S15: HR-DART-MS positive mode ion of compound 2.

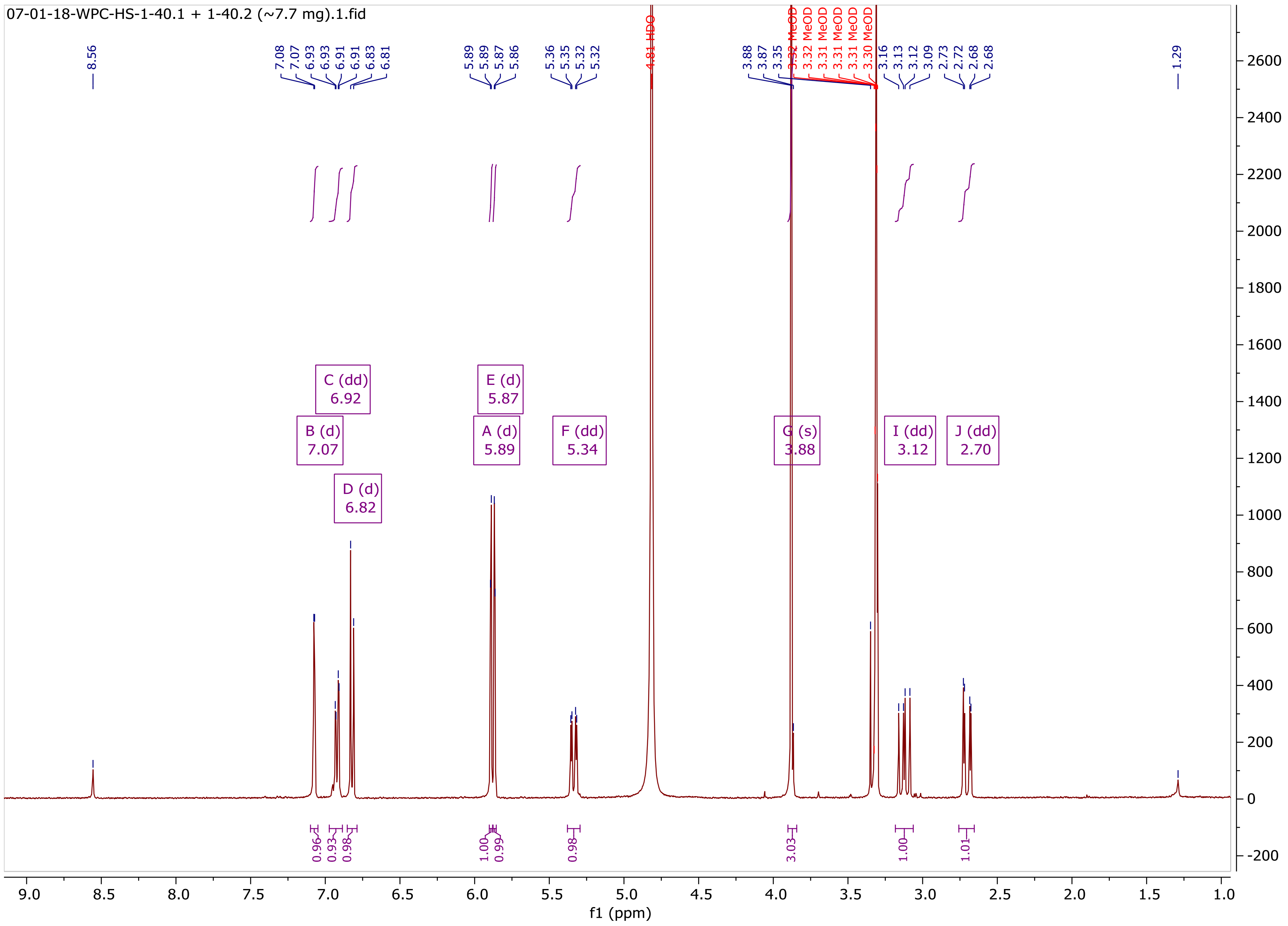

Figure S16: ^{1}H-NMR spectrum of compound 2.

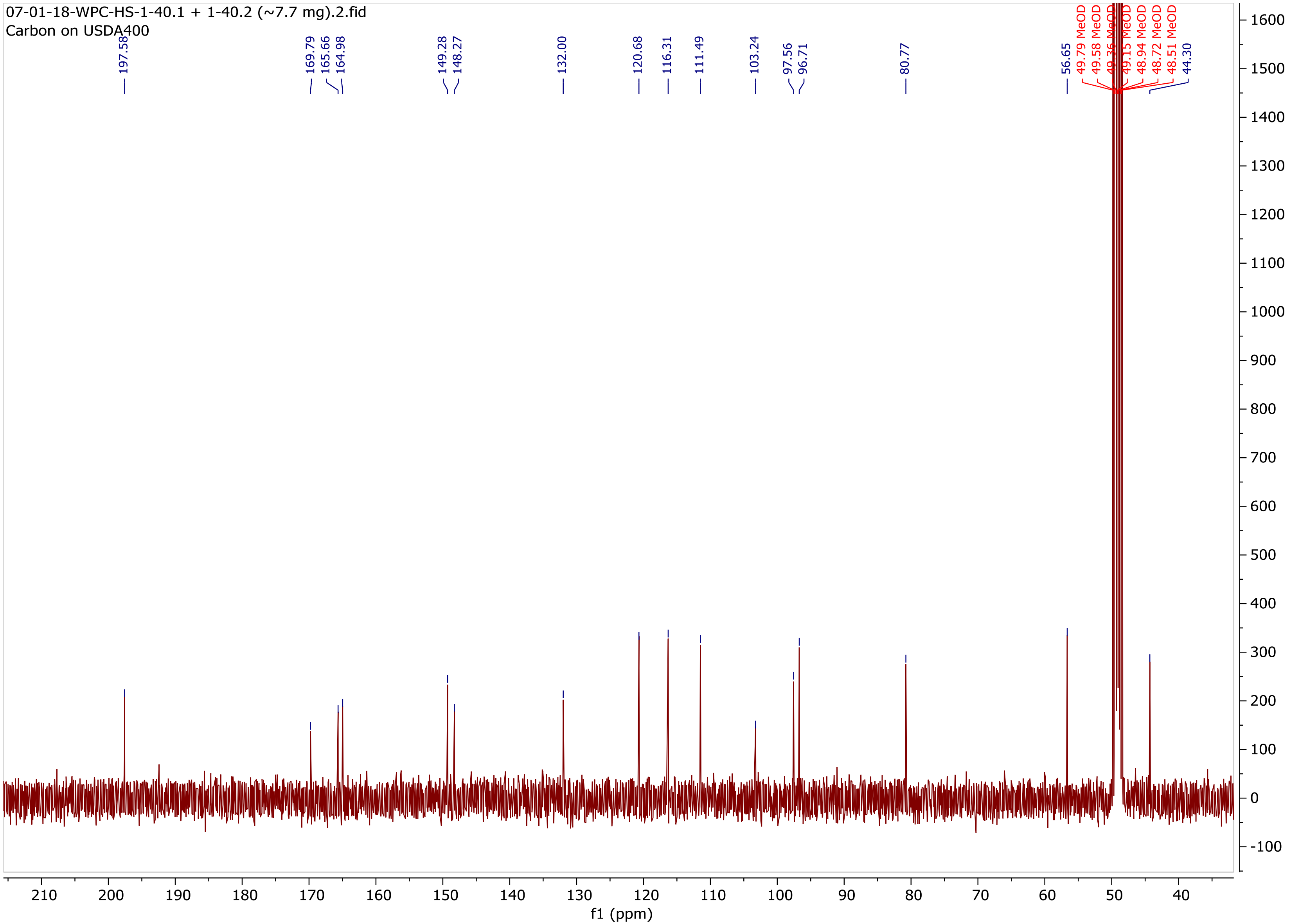

Figure S17: ^{13}C-NMR spectrum of compound 2.

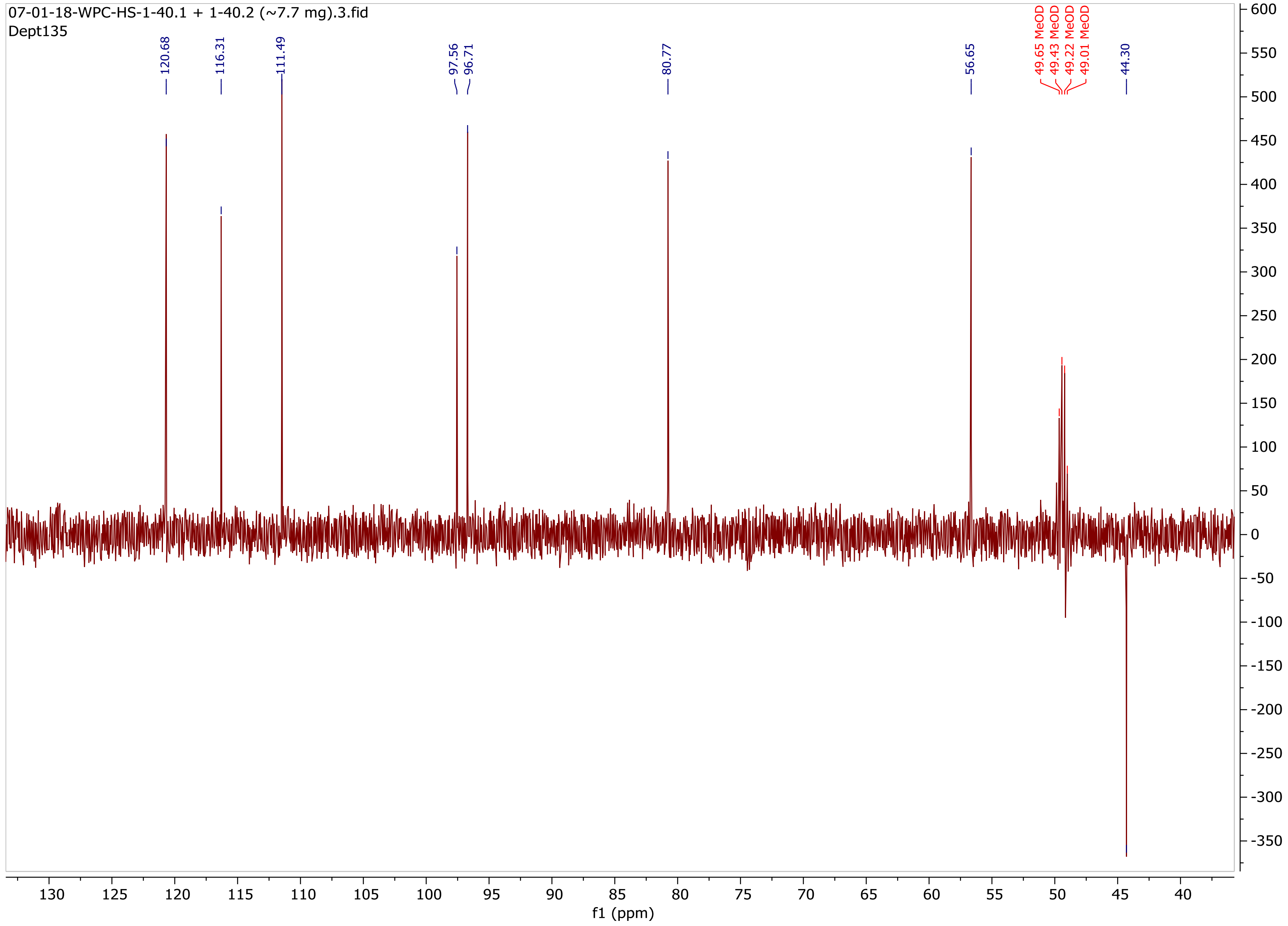

Figure S18: DEPT 135 spectrum of compound 2.

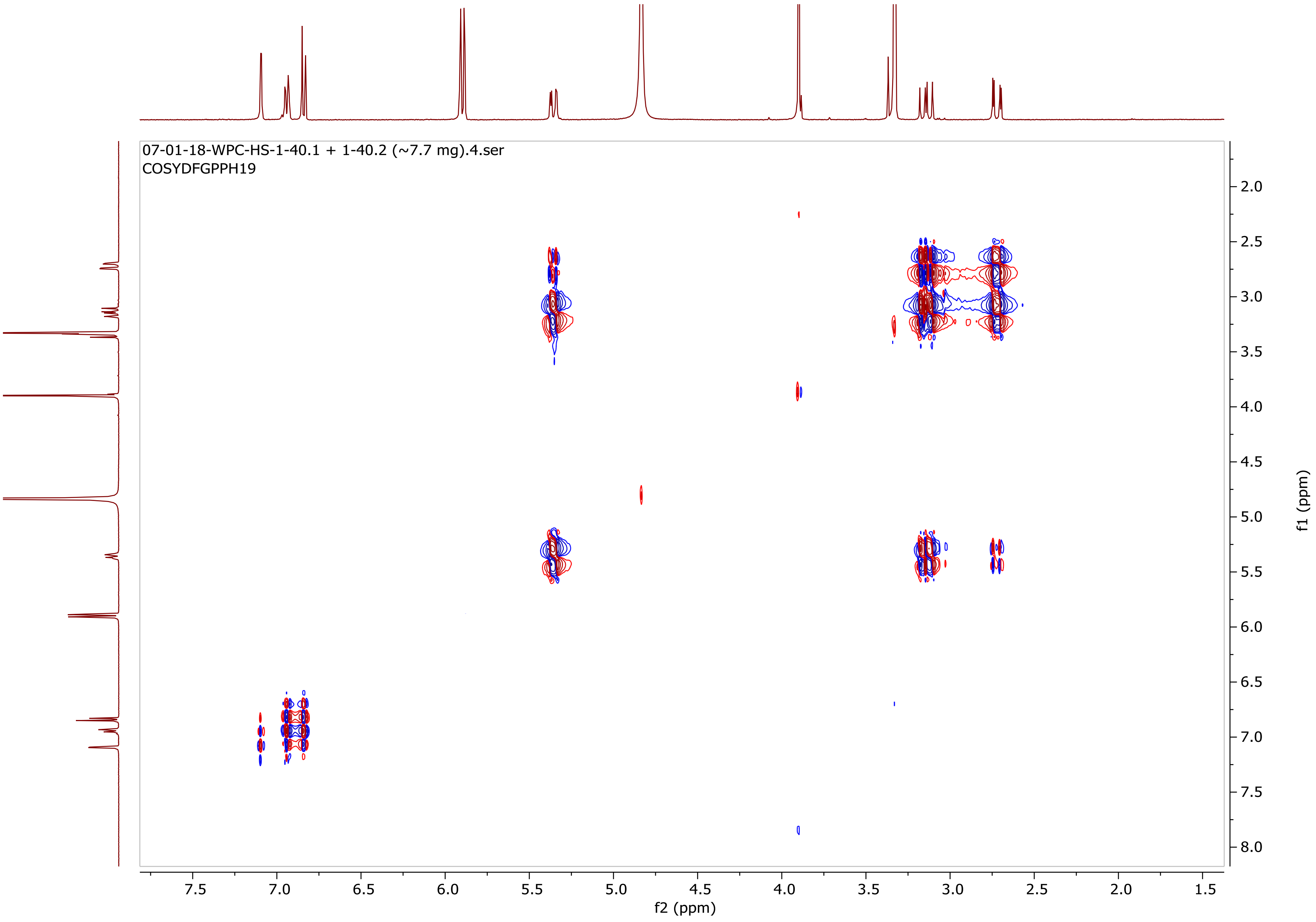

Figure S19: COSY spectrum of compound 2.

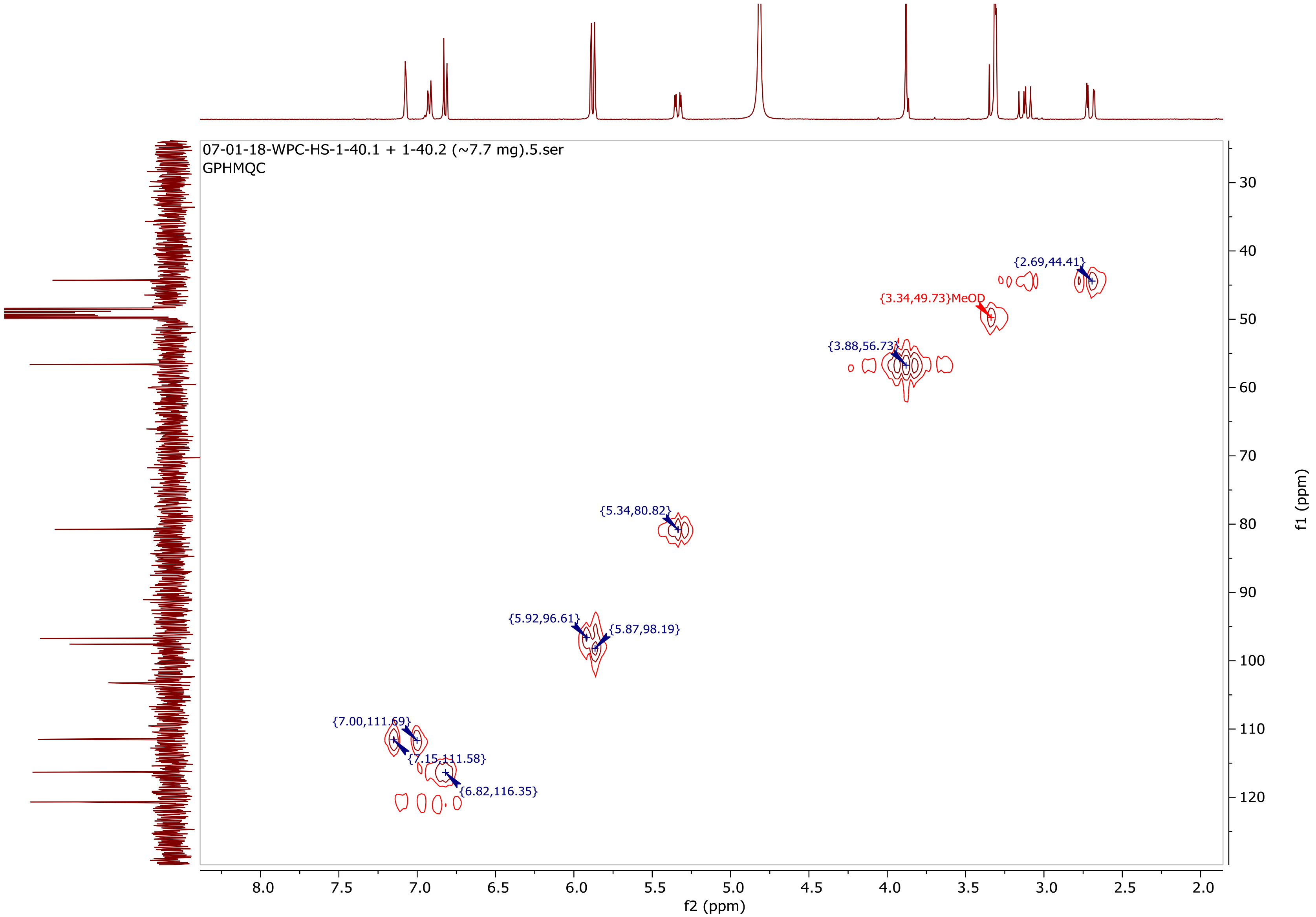

Figure S20: HMQC spectrum of compound 2.

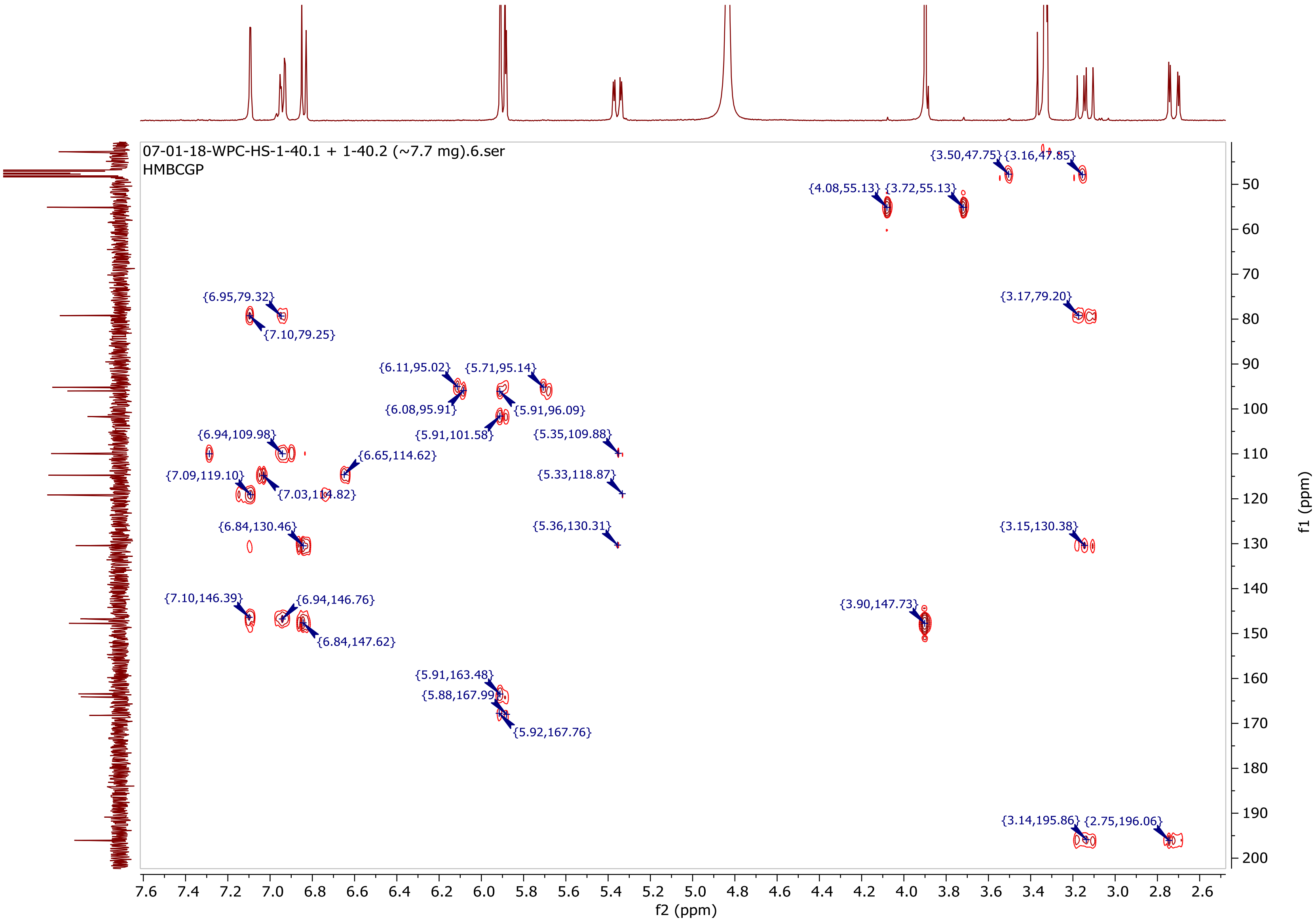

Figure S21: HMBC spectrum of compound 2.

Compound 3 (Salsolol C)

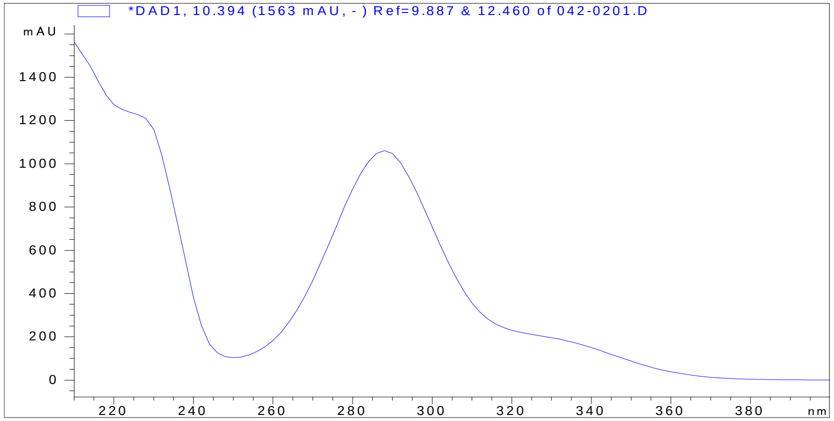

Figure S22: UV spectrum of compound 3.

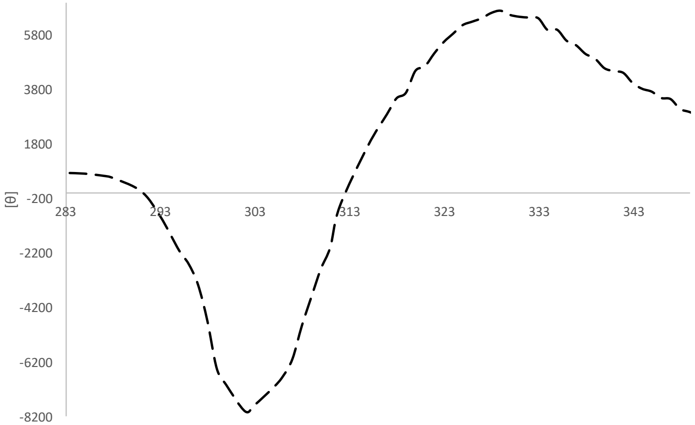

Figure S23: CD spectrum of compound 3.

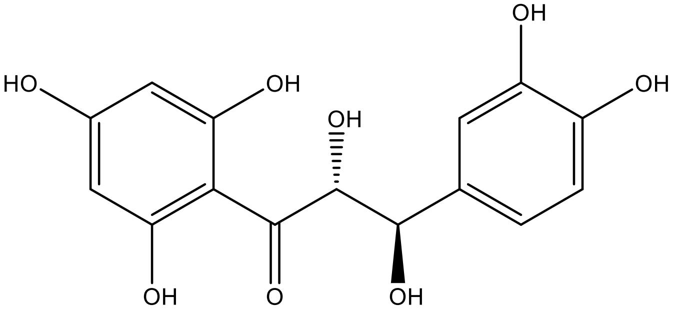

Chemical Formula: $C_{15}H_{14}O_8$
Molecular Weight: 322.27

Figure S24: Chemical structure of compound 3.

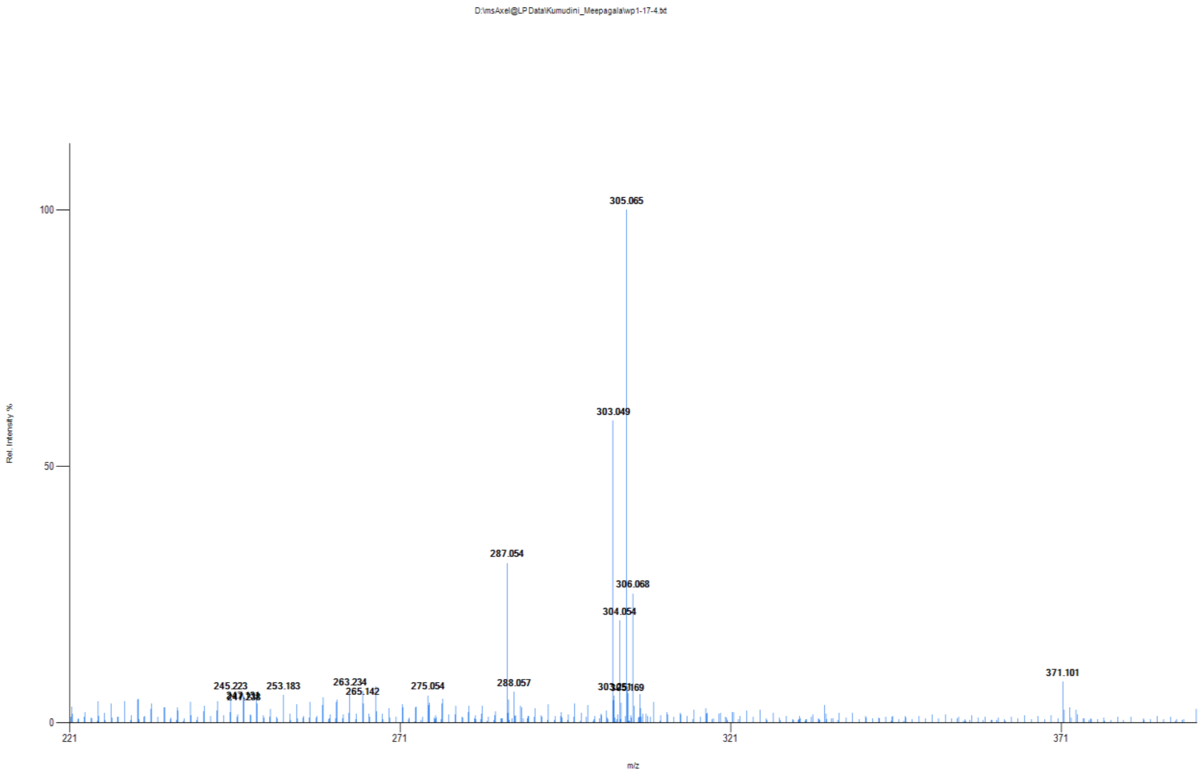

Elemental Compositions

Element Limits: C 0/50 H 0/100 O 0/10

Tolerance: 7 mmu   Even or odd electron ion or both: Both

Electron correction: None.   Charges: 1

Minimum unsaturation: -1   Maximum unsaturation: 100

Calc. m/z	Abund %	mmu	Peaks	Score	DBE	Composition	NIST
305.066128	1.07	0.63	4	0.016950	9.5	C15H13O7	0

Figure S25: HR-DART-MS positive mode ion of compound 3.

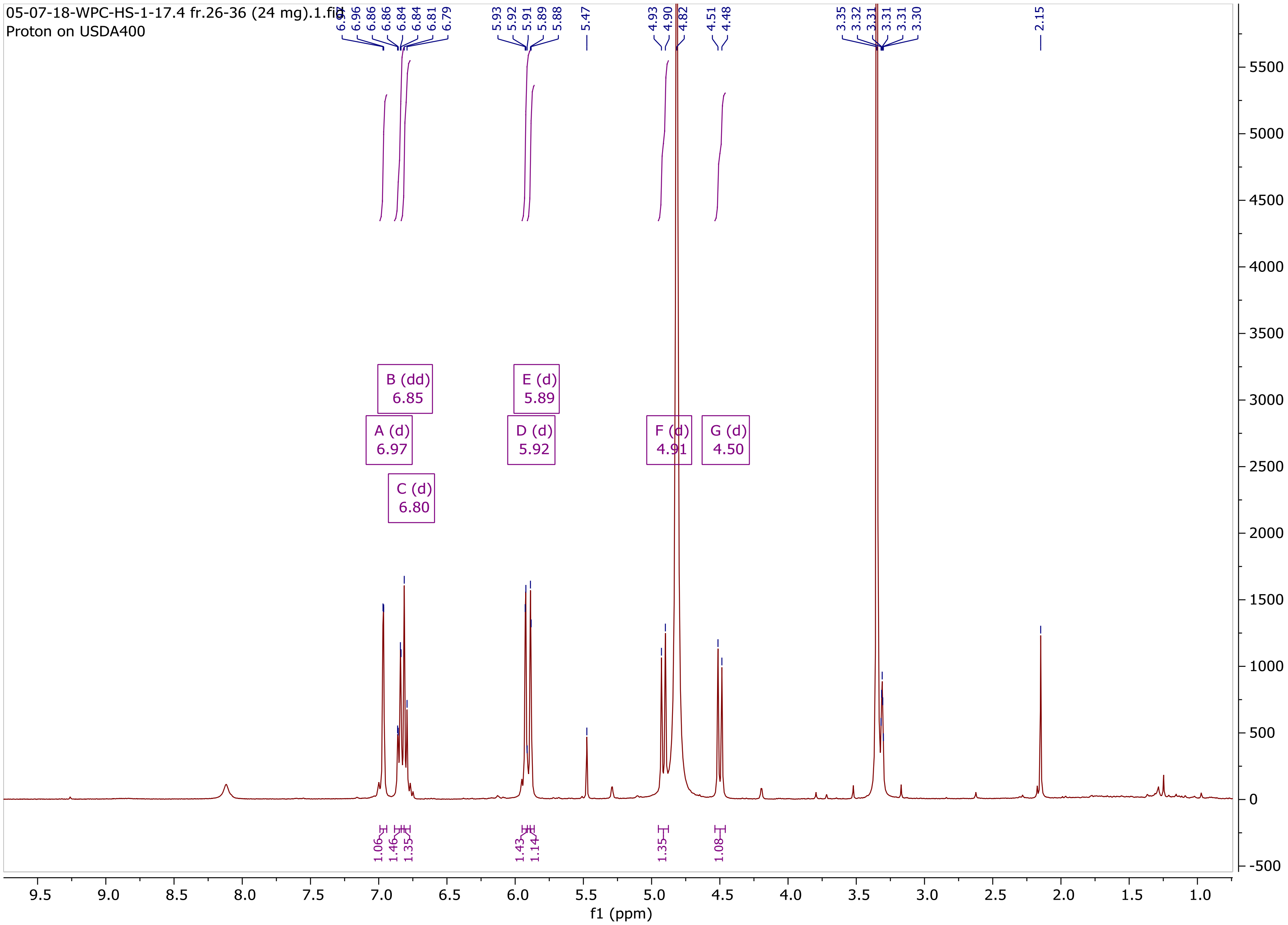

Figure S26: ^{1}H-NMR spectrum of compound 3.

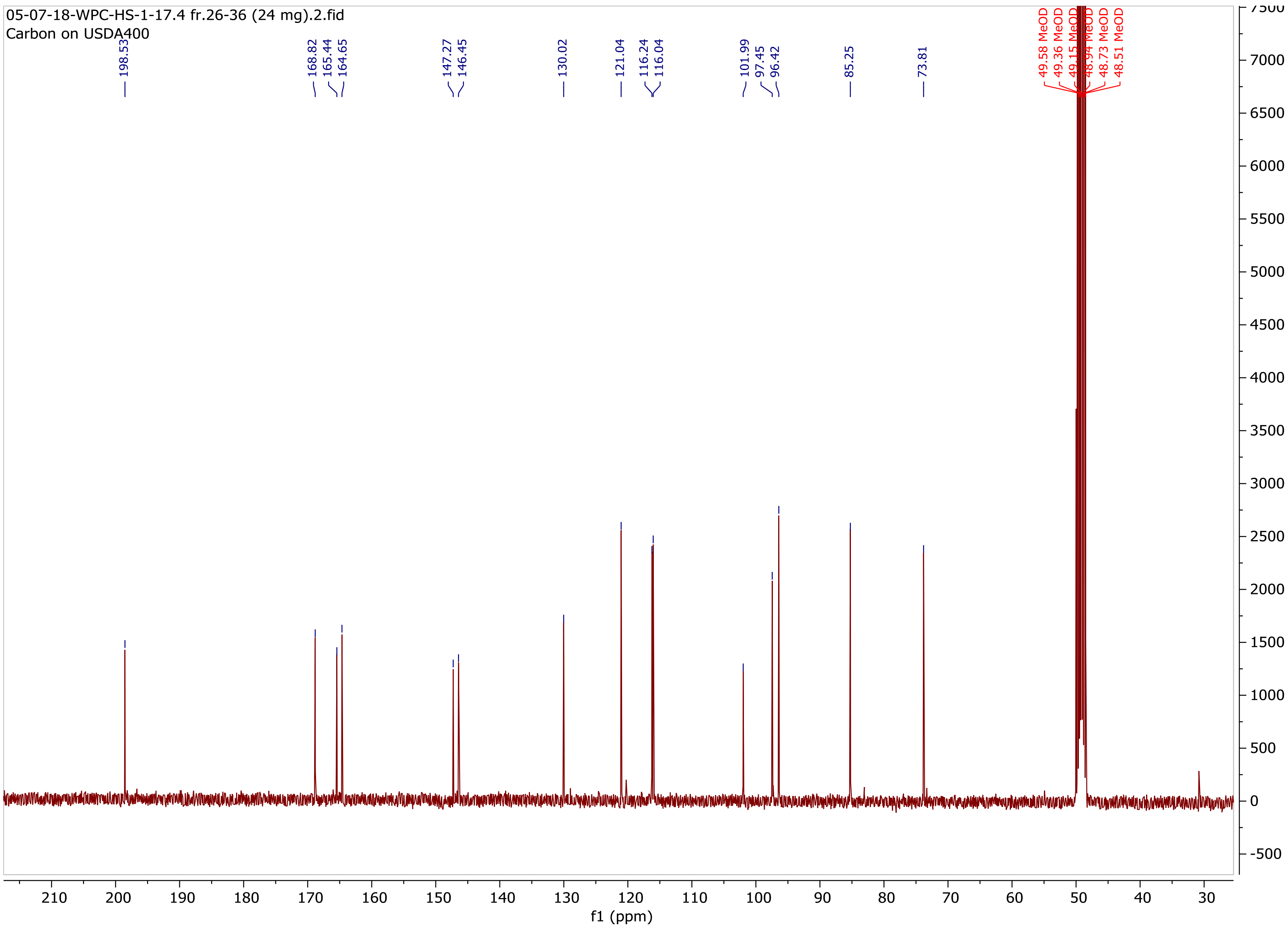

Figure S27: ^{13}C-NMR spectrum of compound 3.

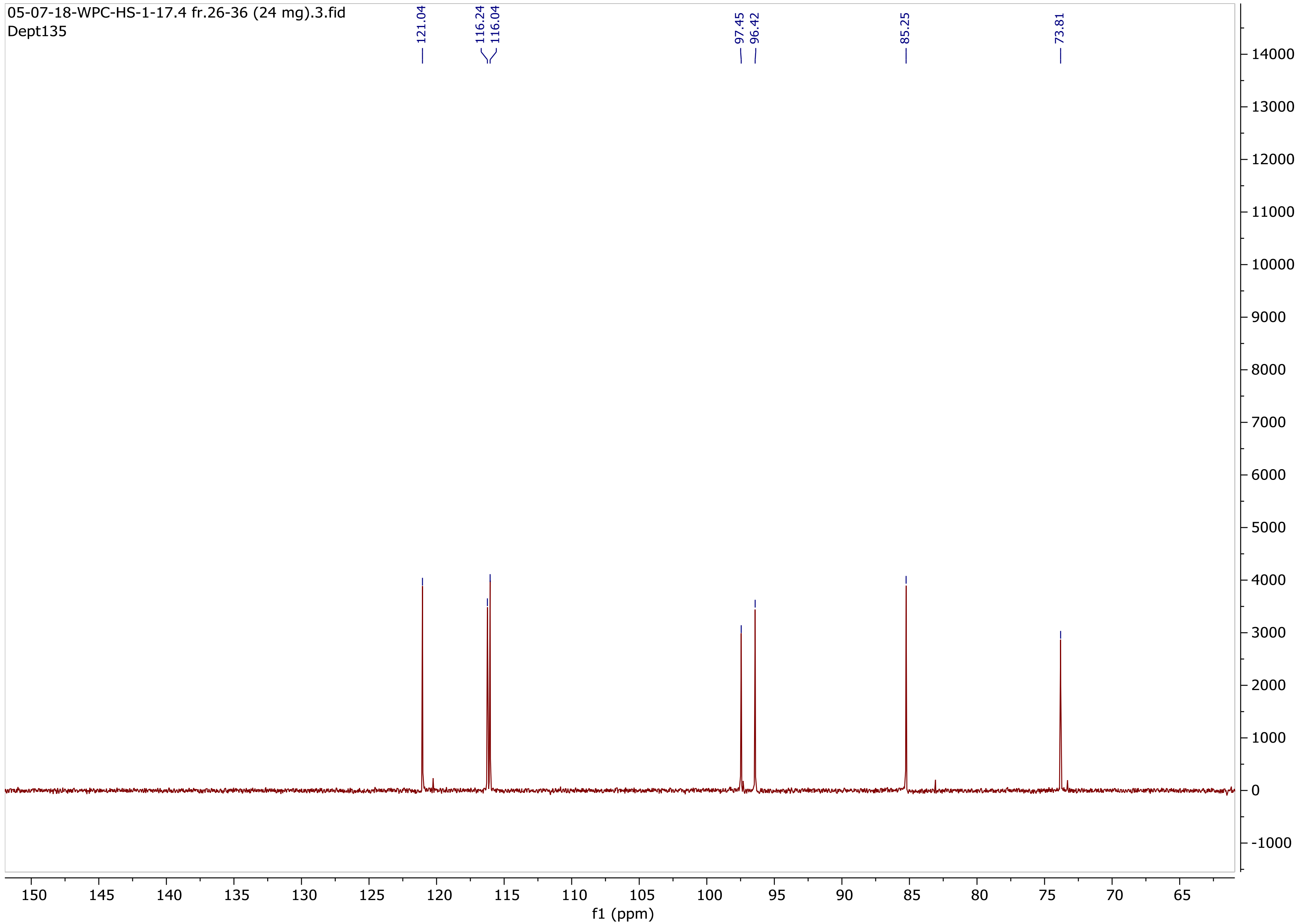

Figure S28: DEPT 135 spectrum of compound 3.

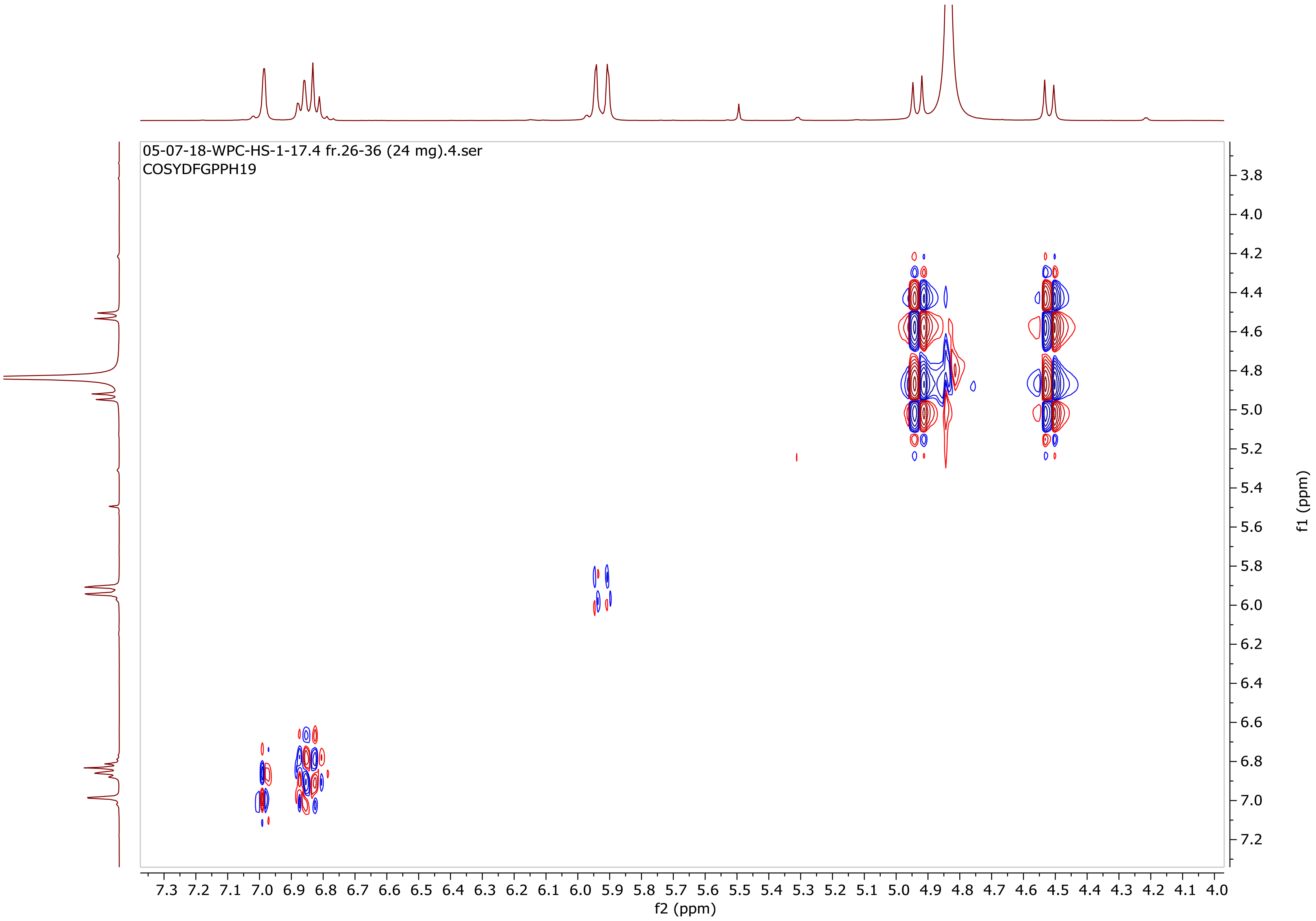

Figure S29: COSY spectrum of compound 3.

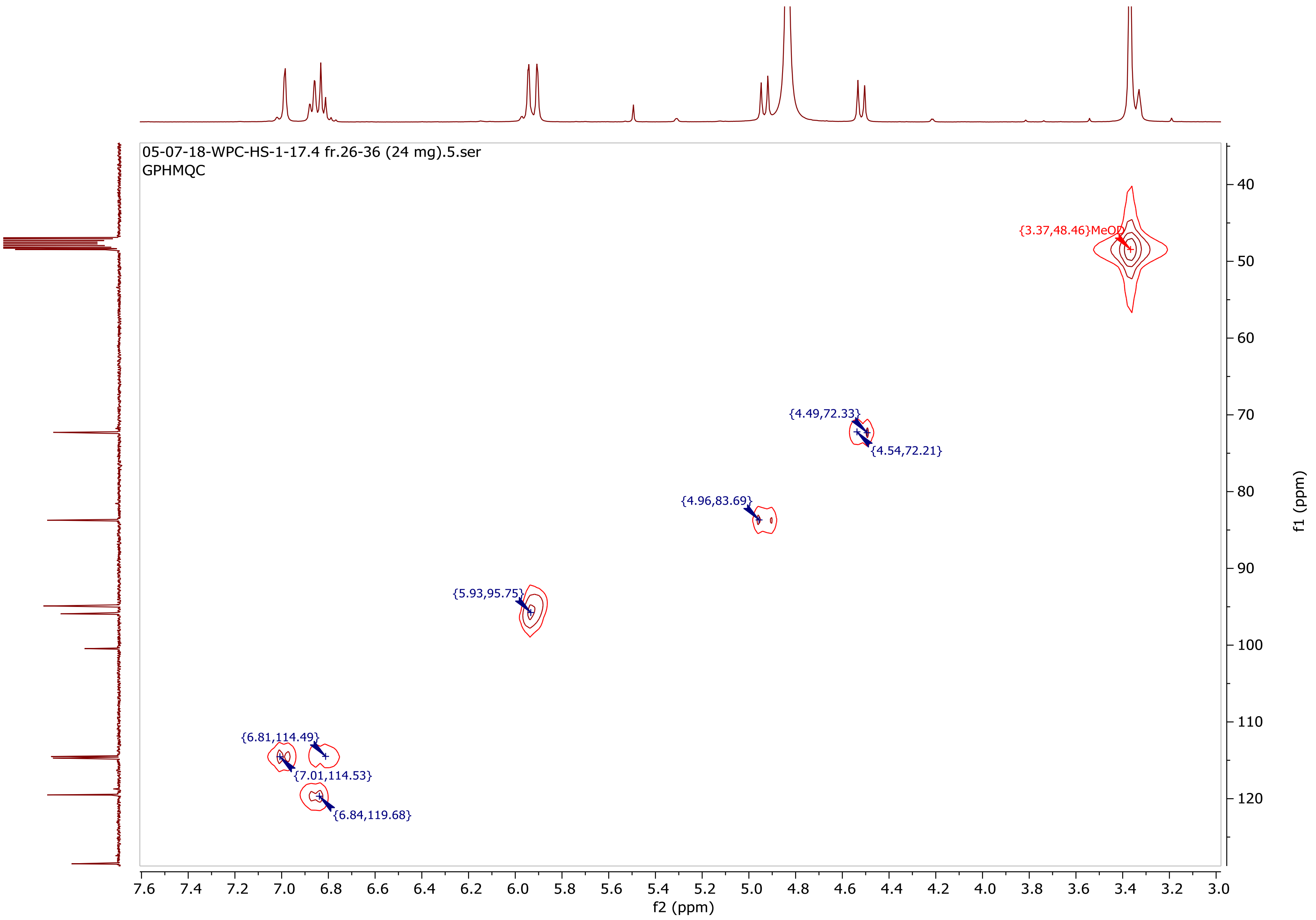

Figure S30: HMQC spectrum of compound 3.

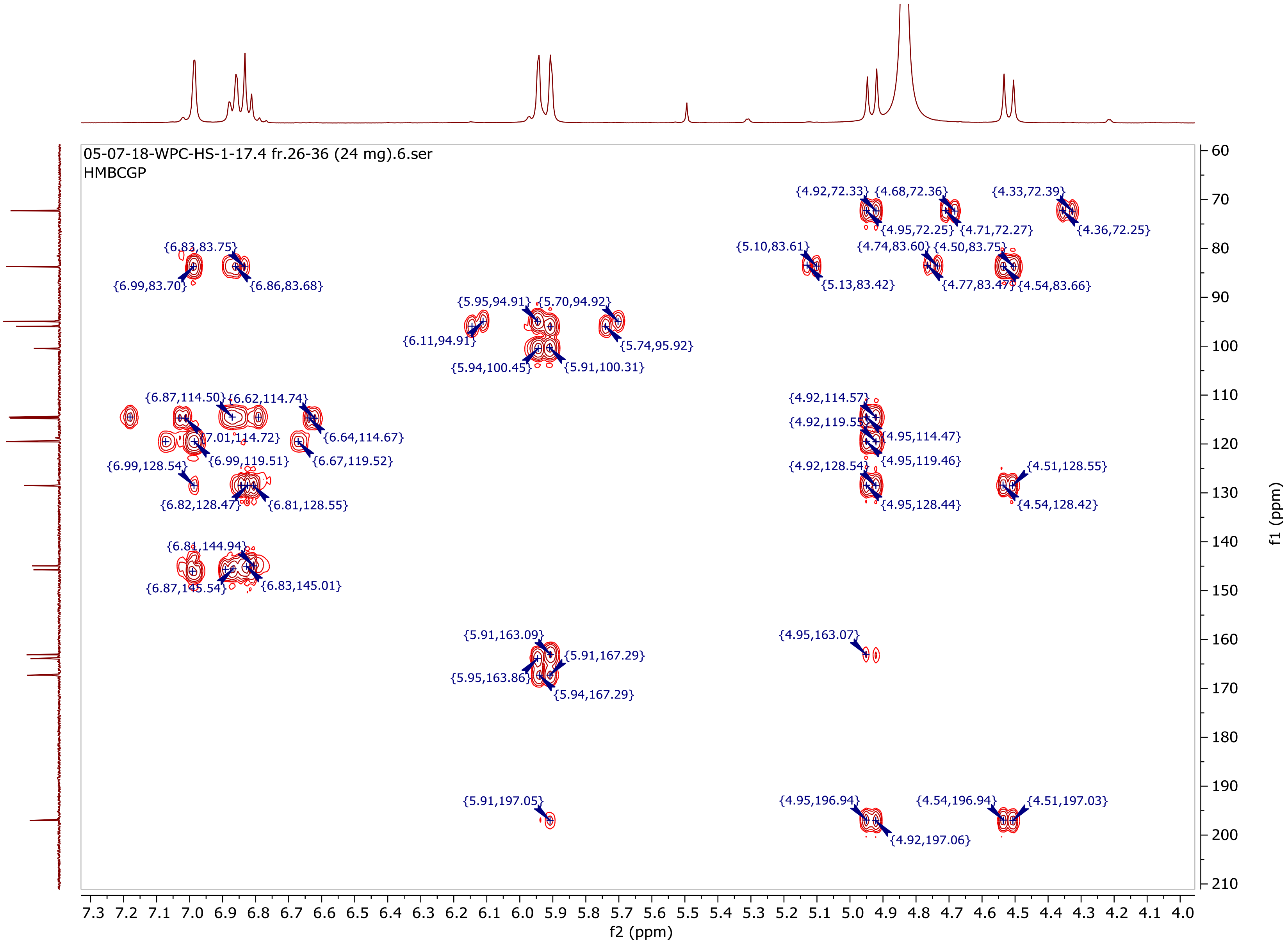

Figure S31: HMBC spectrum of compound 3.

Compound 4 (Balanochalcone)

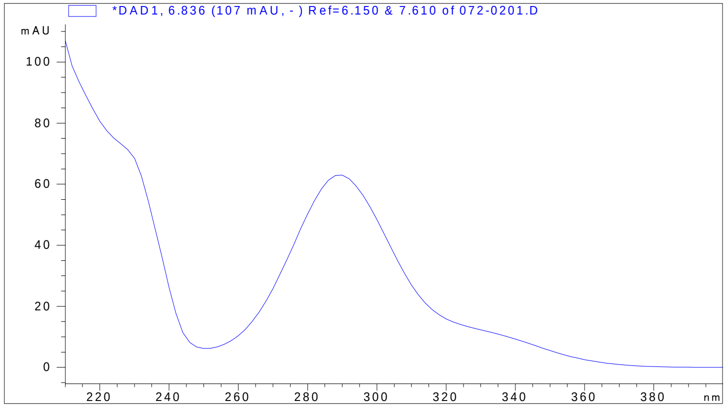

Figure S32: UV spectrum of compound 4.

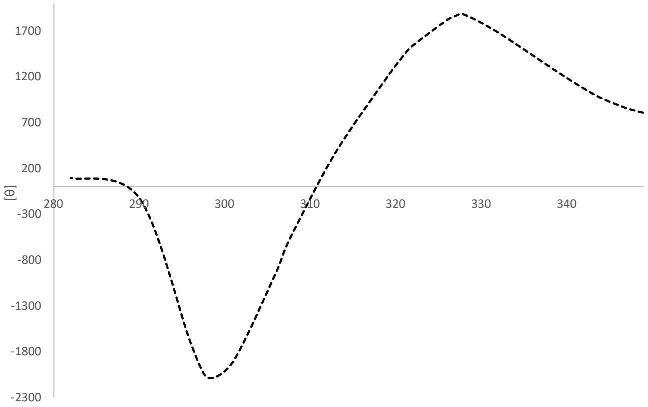

Figure S33: CD spectrum of compound 4.

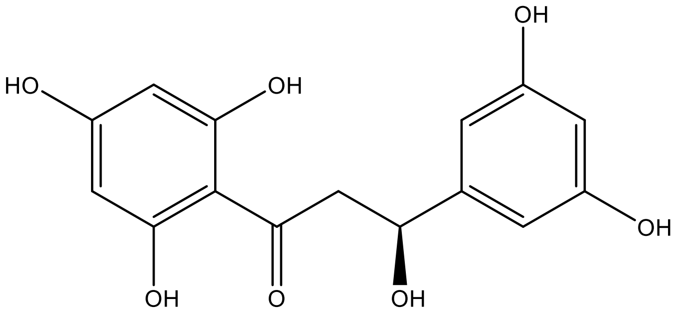

Chemical Formula: $C_{15}H_{14}O_7$
Molecular Weight: 306.27

Figure S34: Chemical structure of compound 4.

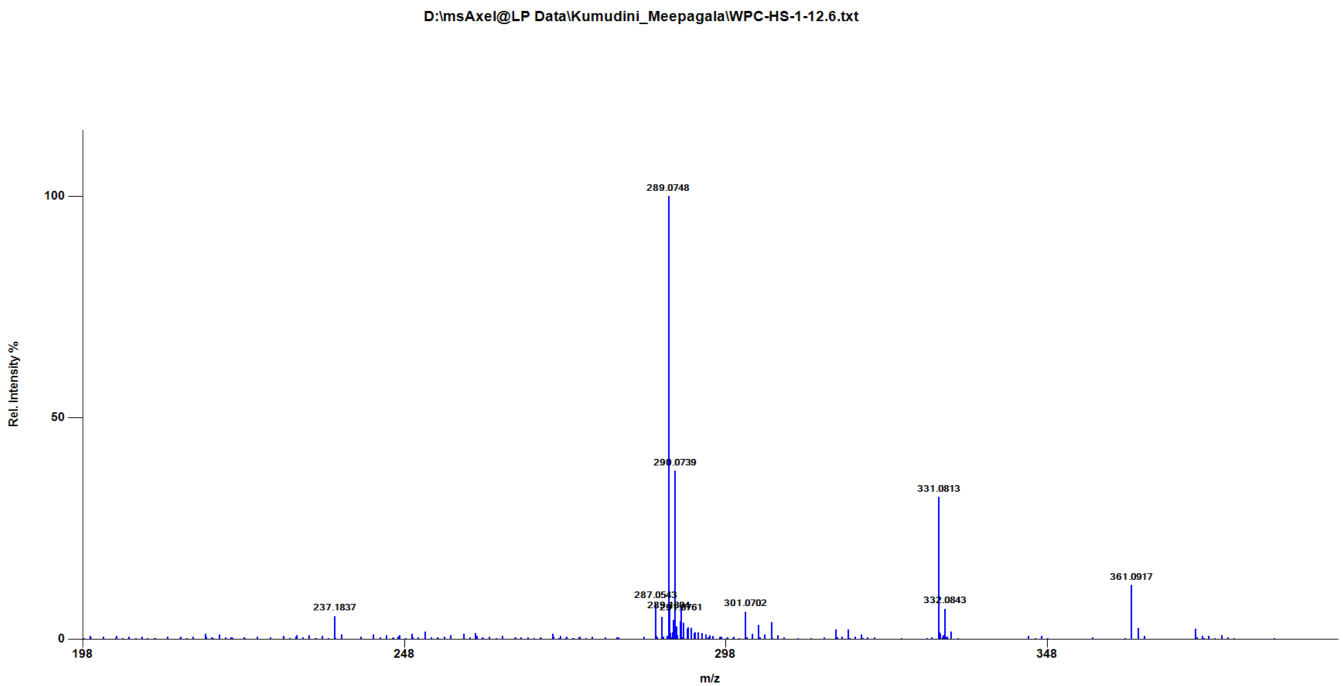

Elemental Compositions

Element Limits: C 0/50 H 0/100 O 0/10

Tolerance: 5 mmu Even or odd electron ion or both: Both

Electron correction: None. Charges: 1

Minimum unsaturation: -1 Maximum unsaturation: 100

Calc. m/z	Abund %	mmu	Peaks	Score	DBE	Composition	NIST
289.071213	4.04	1.81	4	0.183338	9.5	C15H13O6	0

Figure S35: HR-DART-MS positive mode ion of compound 4.

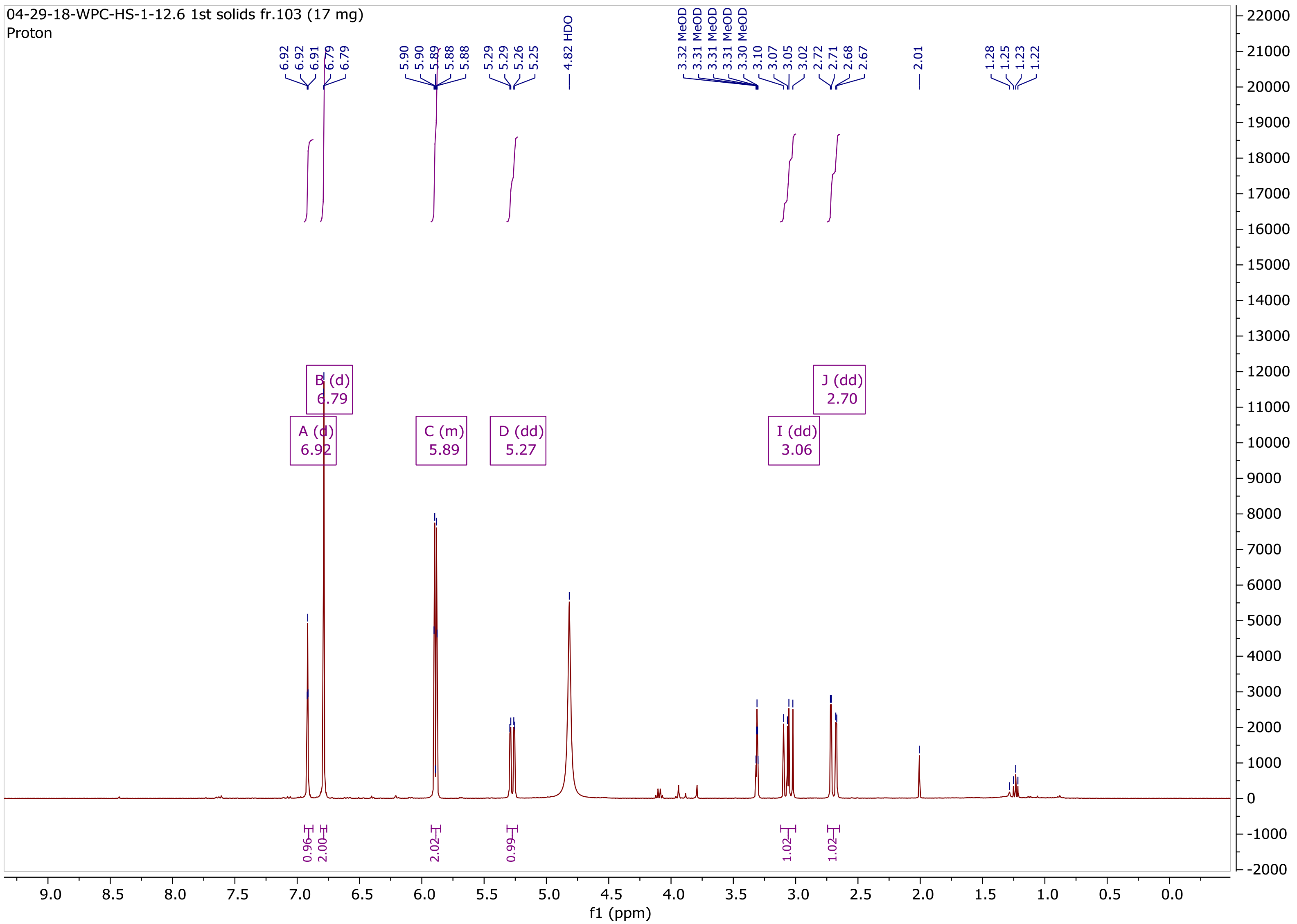

Figure S36: ^{1}H-NMR spectrum of compound 4.

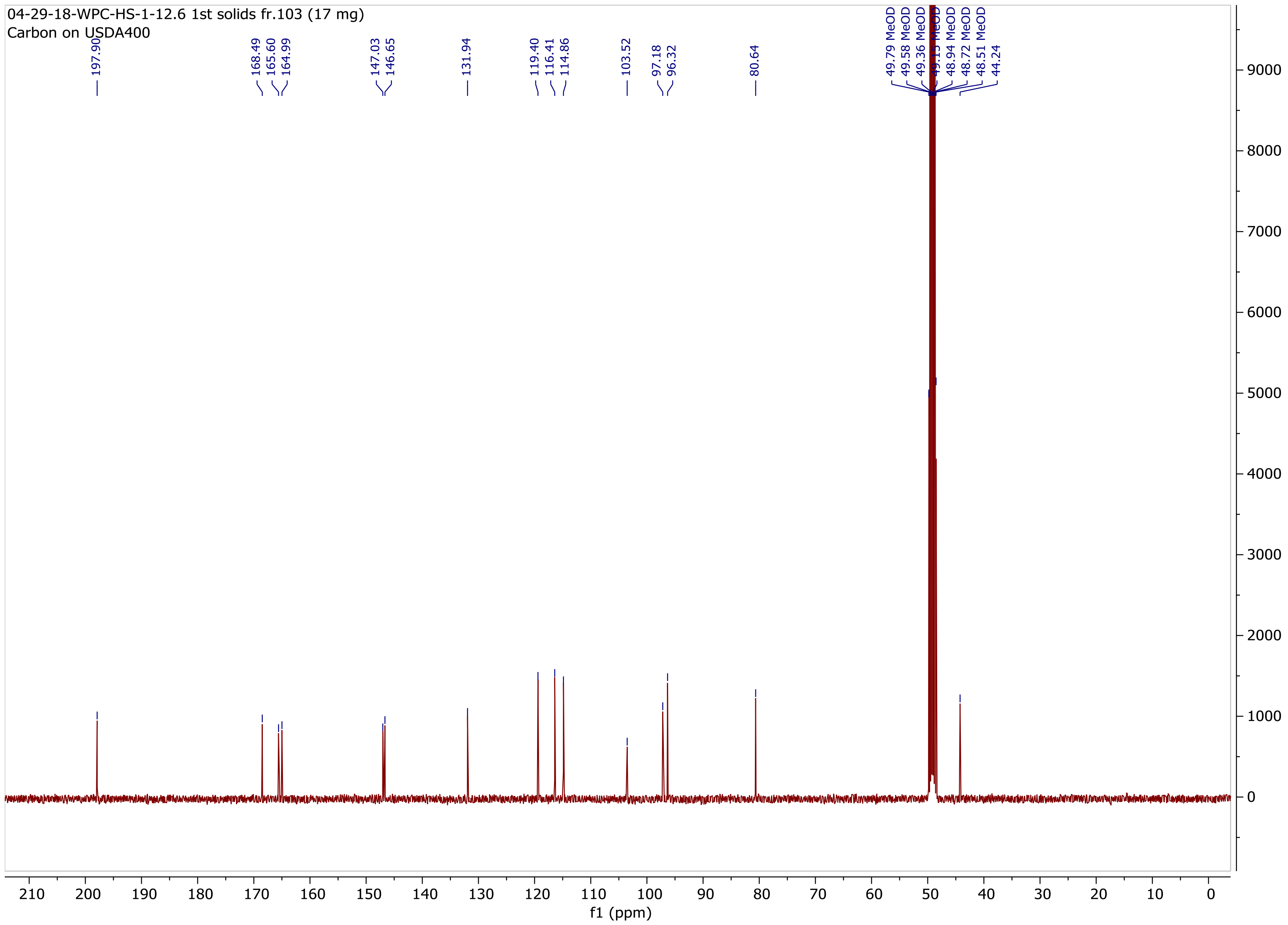

Figure S37: ^{13}C-NMR spectrum of compound 4.

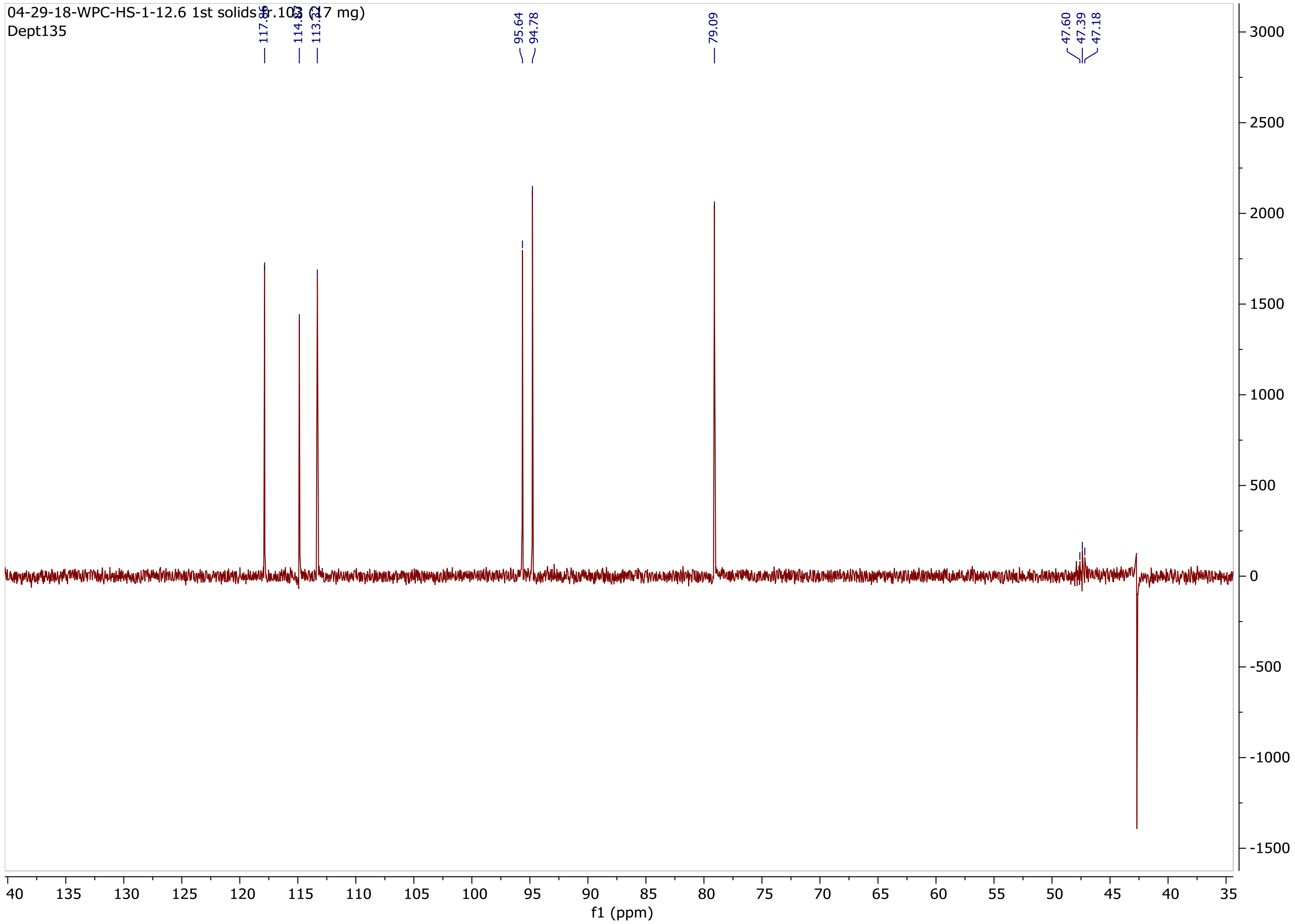

Figure S38: DEPT 135 spectrum of compound 4.

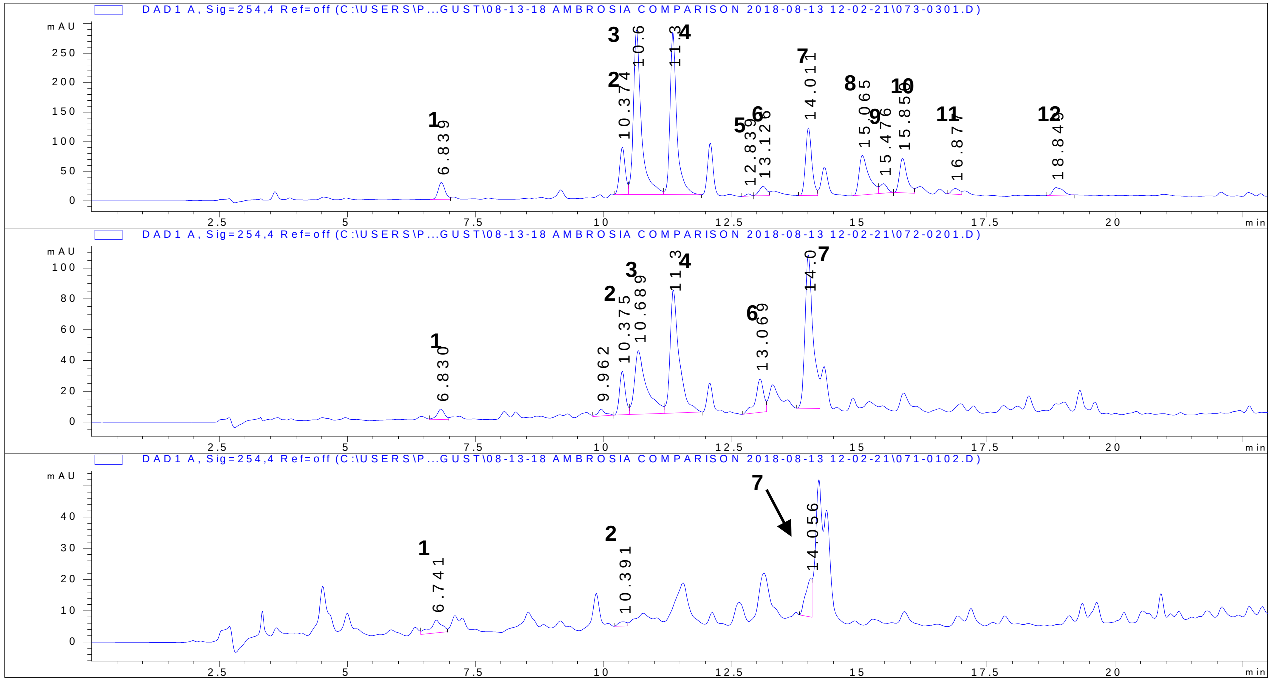

Figure S39: HPLC chromatograms of ethyl acetate extracts (10 mg/mL) recorded at 254 nm for *A. salsola* collected in Texas **(A)**, *A. salsola* from Arizona **(B)** and *A. dumosa* **(C)**. Salsolol C **(1)**, balanochalcone **(2)**, quercetin **(3)**, quercetin 3-methylether **(4)**, salsolol B **(5)**, salsolol A **(6)**, quercetin 3,7-dimethylether **(7)**, quercetin 7-methylether **(8)**, confertin **(9)**, quercetin 3,4'-dimethylether **(10)**, neoambrosin **(11)** and quercetin 3,4',7-trimethylether **(12)**
